# A remarkable sample of bird teeth from the palaeo-Arctic Prince Creek Formation of northern Alaska elucidates Late Cretaceous avialan richness

**DOI:** 10.1098/rsos.261403

**Published:** 2026-10-21

**Authors:** Lauren N. Wilson, John P. Wilson, Daniel T. Ksepka, Caleb M. Brown, Gregory M. Erickson, Donald B. Brinkman, Jaelyn J. Eberle, Patrick S. Druckenmiller

**Affiliations:** ^1^ Earth Sciences, University of Alaska Museum of the North, Fairbanks, AK, USA; ^2^ Geosciences, University of Alaska Fairbanks, Fairbanks, AK, USA; ^3^ Geosciences, Princeton University, Princeton, NJ, USA; ^4^ Bruce Museum, Greenwich, CT, USA; ^5^ Royal Tyrrell Museum of Palaeontology, Drumheller, Alberta, Canada; ^6^ Biological Science, Florida State University, Tallahassee, FL, USA; ^7^ Earth Science and Museum of Natural History, University of Colorado, Boulder, CO, USA

**Keywords:** Avialae, Late Cretaceous, teeth, palaeo-Arctic, birds, Aves, Crocodyliformes, polar

## Abstract

Understanding the taxonomic affinity of putative avialan teeth is essential to assessing the geographic range and richness of Late Cretaceous toothed birds. Isolated teeth attributed to Avialae are known from numerous North American Late Cretaceous microfossil assemblages. However, because only a select few of these tooth types are known from teeth preserved in jaws, their taxonomic affinities remain uncertain. Several key morphologies used to diagnose avialan teeth are also present in non-avialan theropods and crocodyliforms, leading some to question whether many such isolated teeth belong to birds. Here, we provide detailed descriptions and high-resolution images of avialan teeth from the Late Cretaceous Prince Creek Formation (PCF) of northern Alaska. A total of 105 isolated teeth were identified as avialan and can be divided into seven distinct morphotypes. Crucially, decades of extensive microfossil sampling have failed to produce any evidence of crocodyliforms in the PCF, allowing us to rule out this possible identity. Further, the teeth described here can be distinguished from those of all non-avialan theropods known in the PCF. Our morphotypes thus provide an important framework for identifying bird teeth in other Late Cretaceous microfossil assemblages as well as understanding avialan diversity before the K–Pg extinction.

## 1. Introduction

Apart from associated skeletons of hesperornithines and *Ichthyornis* [1–3], Late Cretaceous avialan assemblages of North America consist primarily of isolated, fragmentary postcranial elements and numerous referred isolated teeth [4–9]. Although crown birds (Neornithes) are entirely edentulous and possess rhamphothecae, Mesozoic stem birds typically possessed teeth in at least some portions of their jaws [10,11]. *Hesperornis* and *Ichthyornis* teeth preserved within jaws provide our best reference framework for how tooth morphology varies across the dentition of near-crown stem birds [12]. However, most other North American putative avialan teeth, especially those from terrestrial formations, are neither preserved within jaws nor firmly associated with skeletal remains, leaving their taxonomic affinity ambiguous. Lacking an understanding of such teeth precludes an accurate assessment of the evolutionary history and diversification of Late Cretaceous birds in North America, especially in light of a fragmentary skeletal fossil record. By extension, our inability to identify such teeth to the species level, or at least morphotype, risks underestimating taxonomic richness and the true extinction rate among birds across the Cretaceous–Palaeogene (K–Pg) mass extinction.

Peng *et al.* [13] were the first to identify isolated teeth as ‘Aves indet.’ on the basis of clear differences from other known theropod teeth. These 27 teeth came from the Foremost and Oldman formations of southeastern Alberta and were described as follows: (i) small; (ii) stout but strongly laterally compressed; (iii) possessing a slightly recurved crown; (iv) enamel carinae on both the anterior and posterior edges; (v) lacking definitive denticles; and (vi) prominently constricted at the base of the crown. Sankey *et al.* [14] continued this trend shortly thereafter, identifying teeth from the Campanian Judith River Formation (Belly River Group) as ‘Aves indet.’ based on similarities to *Hesperornis* teeth in jaws [14] and noting similar features to those described by Peng *et al.* [13]. However, some non-avialan taxa, including crocodyliforms, also possess at least some of these features, particularly labiolingual compression and basal root constriction, which could result in the misidentification of isolated teeth. This similarity was noted by Peng *et al.* [13], who indicated that the small teeth of *Leidyosuchus* could be confused with ‘Aves indet.’ teeth, but that they remain separable on the basis of the greater degree of lateral compression and stronger distal recurvature in avialan teeth.

Following these initial studies, a diversity of similar, isolated, putative avialan teeth have been recorded from microsite material from a variety of Late Cretaceous lithostratigraphic units in western North America, including the Aguja and Javelina formations of Texas, Straight Cliffs Formation of Utah, Lance Formation of Wyoming, Hell Creek Formation of Montana and Milk River, Foremost, Oldman, Dinosaur Park, St Mary River, Wapiti and Horseshoe Canyon formations of Alberta [13–20]. Crucially, these units span geographically from southern Texas to northern Alberta and temporally from the Santonian (and possibly Cenomanian to early Turonian [21]) to the late Maastrichtian, indicating a wide geographic and temporal range.

Longrich [22] described additional putative avialan teeth from the Lance Formation of Wyoming but noted that the teeth were potentially too large to be avialan (crown height: 6 mm) and may therefore instead be crocodyliform; however, he noted that they were more laterally compressed than expected for a crocodyliform. Mohr *et al.* [23] expanded upon this possibility, hypothesizing that the majority of ‘cf. Aves’ teeth from the terrestrial Late Cretaceous formations of Alberta represented immature or dwarf crocodyliforms. Given the complications that accompany identifying isolated teeth, such as the presence of similar features in multiple taxa or families (e.g. ecologically driven homoplasy), tooth row variation and ontogenetic change [17,24], it becomes vital to carefully consider all possible taxonomic identities.

Teeth exhibiting typical ‘cf. Aves’ morphologies are consistently recovered in microfossil assemblages from multiple North American Late Cretaceous terrestrial microfossil sites [14,18,22,23,25] that include skeletal material from ectothermic tetrapods, including lissamphibians, squamates, turtles and, most crucially, crocodyliforms [26]. The Late Cretaceous Prince Creek Formation (PCF) of the North Slope of Alaska (figure 1A) provides a unique opportunity to analyse Mesozoic ecosystems that completely lack terrestrial ectothermic taxa. After nearly four decades of study, the absence of fossils from any ectothermic tetrapods strongly suggests that they were unable to tolerate the extreme winter conditions (sub-freezing temperatures and several months of continuous darkness) that prevailed at the high palaeolatitudes (80–85° N) where the PCF was deposited [27,29,30]. The PCF also preserves one of the richest assemblages of Late Cretaceous avialan skeletal fossils, including representation of Ichthyornithes, Hesperornithes and near-crown or crown birds [31]. Here we describe a sample of 105 isolated teeth from the PCF that possess similar morphologies to teeth from penecontemporaneous formations previously attributed to ‘cf. Aves’. We identify seven discernible morphotypes that are described in detail below. Based on the well-established absence of ectothermic tetrapods in the PCF, and four decades of extensive sampling with no fossil evidence of crocodyliforms, we confidently determine that these teeth are not referable to crocodyliforms. The teeth are also not referable to any of the known, adult or perinatal, toothed PCF non-avialan theropods, which include Tyrannosauridae, Troodontidae and Dromaeosauridae [27]. Thus, we infer that the most parsimonious conclusion is that these isolated teeth belong to avialans, contributing a dental fossil record complimentary to the PCF avialan body fossil record [31].

**Figure 1.**
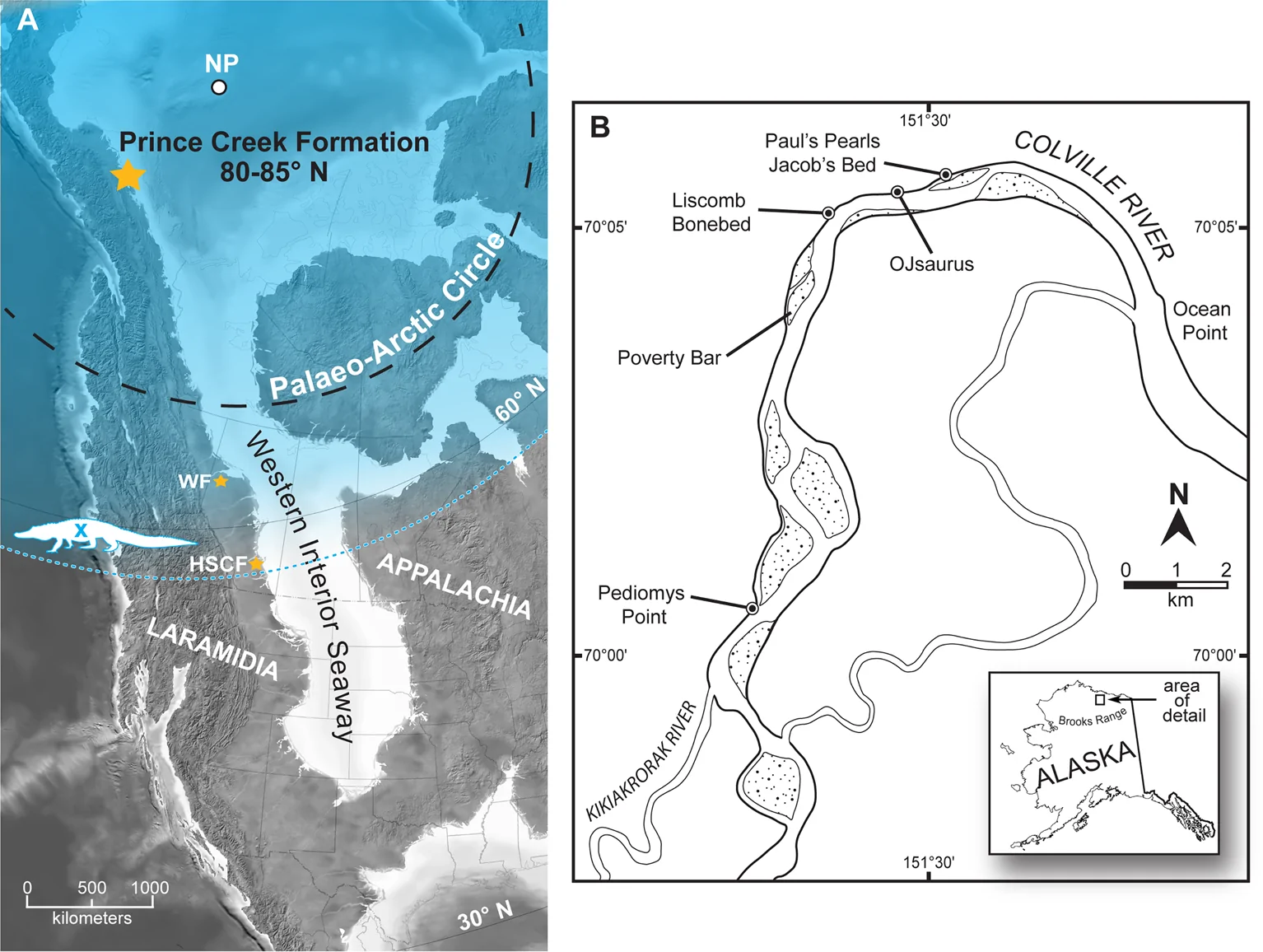
(A) North American palaeogeography 72 Ma, map by Ron Blakey. Figure modified with permission from Druckenmiller *et al.* [27]. Dashed line indicates the latitudinal boundary above which crocodylian fossils are rare to absent. (B) Maps showing the location of the four vertebrate microfossil localities that yielded the material described in this paper. Figure modified with permission from Brinkman *et al.* [28]. HSCF, Horseshoe Canyon Formation; NP, North Pole; WF, Wapiti Formation.

We enlist non-destructive, high-resolution scanning electron microscopy (SEM) techniques to image the PCF avialan teeth. This imaging technique eliminates common issues when visualizing small teeth, including intense light glare that can obscure details such as carinae morphology and enamel ornamentation. In contrast to typical SEM techniques used when imaging isolated teeth, we enlist a completely reversible and non-destructive mounting strategy that allows for imaging of millimetre-scale teeth without damage to the specimens or application of difficult-to-remove substances. This visualization method allows for comparative studies with lower-latitude crocodyliform-bearing Late Cretaceous assemblages, increasing our knowledge of the distribution and richness of avialans preceding the K–Pg mass extinction.

*Institutional abbreviations*: UAMES, University of Alaska Museum of the North Earth Sciences Collection, Fairbanks, Alaska, USA; TMP, Royal Tyrrell Museum of Palaeontology, Drumheller, Alberta, Canada; RSM, Royal Saskatchewan Museum, Regina, Saskatchewan, Canada.

## 2. Material and methods

A large sample of 105 isolated avialan teeth from the PCF is described here. This is by far the largest sample of isolated avialan teeth known from any North American lithological unit [19], rivalling the total number of teeth reported from all other units combined. All teeth are reposited in the University of Alaska Museum of the North Earth Sciences Collection.

A table, including specimen numbers, morphotypes, locality, as well as crown height and basal width measurements for the PCF teeth, is available in the supplementary material. Given the level of precision possible with such small teeth, measurements are available for only the teeth that were imaged with the SEM. Basal width was measured as the maximum width at the basalmost extent of the carinae, and crown height was measured as the maximum distance from the basal origin of the carinae to the apical-most point of the crown. All measurements were made in Fiji [32] using the scale from the SEM images.

The PCF avialan teeth come from four microvertebrate assemblages (i.e. microsites), *sensu* [33] (table 1). Microsite sampling in the PCF began over four decades ago. The teeth described here come from one historical site (Pediomys Point) and three more recently discovered localities: Paul's Pearls, Jacob's Bed and OJsaurus (figure 1B). These assemblages are hosted within thin (2–15 cm), silty to sandy, organic-rich lenticular bodies. Collectively, vertebrate fossils preserved in these deposits range from very small teeth (approx. 1 mm) of avialans, fishes, non-avialan dinosaurs and mammals to large, adult dinosaur bones (e.g. hadrosaur tibia). The elements have undergone varying degrees of weathering and transport but typically preserve delicate structures such as tooth roots and articular condyles [27,34].

**Table 1.** Number of avialan teeth from each locality in the PCF.

number of teeth from each locality					
locality	Paul's Pearls	OJsaurus	Jacob's Bed	Pediomys Point	total number
**no. of teeth**	57	1	24	23	105

The specimens described in this study were recovered by screenwashing. Fossiliferous sediment was washed in the lab to remove the clay-size fraction and then screened in water using 2.0 and 0.5 mm screens. The remaining concentrate was examined visually under a microscope, and fossil material was collected. Minor mechanical preparation was performed to clean select specimens for high-resolution imaging. Specimens were mounted to metal imaging stands under magnification using Elmer's washable school glue (Elmer's Products, Westerville, OH, USA), which, unlike other mounting compounds typically used in imaging of fossils (e.g. plasticine clay and certain waxes), is completely water soluble and leaves no residue on the fossil. Unlike other specimen mounting methods, such as clay, in which specimens can break under the pressure of being pressed into the adhesive, no pressure was placed on the fossil when mounting, and the glue released instantly when water was applied after imaging. As such, we also recommend this mounting method for digital photography of microfossils.

Scanning electron microscope images were taken with the University of Alaska Fairbanks Advanced Instrumentation Laboratory's Quanta 200 environmental scanning electron microscope (ESEM; Thermo Fisher Scientific, Waltham, MA, USA) and processed using Adobe Photoshop and Illustrator software (Adobe, San Jose, CA, USA). Conventional SEMs operate in a high-vacuum environment; however, this requires that samples either be highly conductive or permanently mounted and sputter-coated with a highly conductive thin metal film (e.g. 2–4 nm of iridium) to minimize specimen charging and reduce beam penetration, providing a sharper image. To avoid this, we enlisted a low vacuum mode, in which the electron gun chamber remains under high vacuum, but the specimen chamber remains at a lower vacuum (higher pressure), allowing for the investigation of non-conductive specimens without the risk of specimen charging. Precise settings used for each imaged tooth are available as annotations on the raw images included in the Zenodo repository associated with this study [35]. Following imaging, any glue residue was gently washed off with water.

Some debate exists over whether the group that contains *Passer domesticus*, as well as all other extant birds and extinct birds such as *Archaeopteryx* and Enantiornithes, but not bird-like dinosaurs such as *Troodon formosus* and *Dromaeosaurus albertensis*, should be referred to as Aves [36] or Avialae [37,38]. Some researchers reserve Aves for crown birds, using the taxon name Avialae to encompass both crown and stem birds. Other researchers use the name Neornithes to denote crown birds and consider Aves to include all crown and stem birds. As the most recent common ancestor of crown birds is generally agreed to have an edentulous beak [10,39], any fossil bird tooth logically must belong to a stem bird. Therefore, to avoid any ambiguity regarding the use of Aves, we refer all bird teeth to Avialae, regardless of whether prior authors referred them to Aves, and we use Neornithes to refer to crown birds.

Throughout the descriptions, anatomical terminology follows Smith & Dodson [40] and enamel ornamentation terminology follows Hendrickx *et al.* [41].

## 3. Descriptions

Avialan teeth from the PCF were delimited into seven morphotypes based on several consistent features, including degree of recurvature (distal deflection) and curvature (lingual deflection) of the crown, surface enamel ornamentation, basal crown cross-sectional shape, and presence, extent and ornamentation of carinae (table 2; figures 2–9). Lingual and labial surfaces were determined for all teeth under the assumption that labiolingual curvature of the tooth results in lingual deflection (even if very slight). Mesial and distal surfaces were determined under the assumption that the mesiodistal recurvature of the tooth results in distal deflection. Of the 105 teeth analysed, a total of 89 teeth were able to be confidently assigned to one of the seven morphotypes, whereas 16 were too incomplete to assign ( table 3). The abundance of the seven morphotypes is relatively uniform with a maximum count of 17 (morphotypes 1 and 5) and minimum of 7 (morphotype 7).

**Table 2.** Distinguishing features of avialan tooth morphotypes.

morphotype	recurvature (distal)	curvature (lingual)	sulcus	longitudinal ridges	carinae	ornamented carinae
1	moderate	strong	lingual	lingual	prominent	no
2	moderate	strong	lingual	lingual and labial	prominent	no
3	moderate	strong	lingual	lingual and labial	prominent	yes
4	moderate	slight	none	none	moderate	no
5	slight to moderate	little to no	lingual and labial	lingual	moderate	no
6	slight to moderate	little to no	lingual	none	moderate	no
7	slight to strong	little to no	lingual	lingual and labial	moderate	yes

*Notes*: Recurvature refers to the degree of distal deflection. Curvature refers to the degree of lingual deflection. Sulcus refers to the presence or absence of sulci on the lingual and labial faces. Longitudinal ridges refer to the presence or absence of longitudinal ridges on the lingual and labial faces. Carinae refers to the presence and extent of carinae on the mesial and distal tooth margins. Ornamented carinae refers to the presence or absence of ridges on the mesial and distal carinae. See morphotype descriptions for more detailed descriptions.

**Figure 2.**
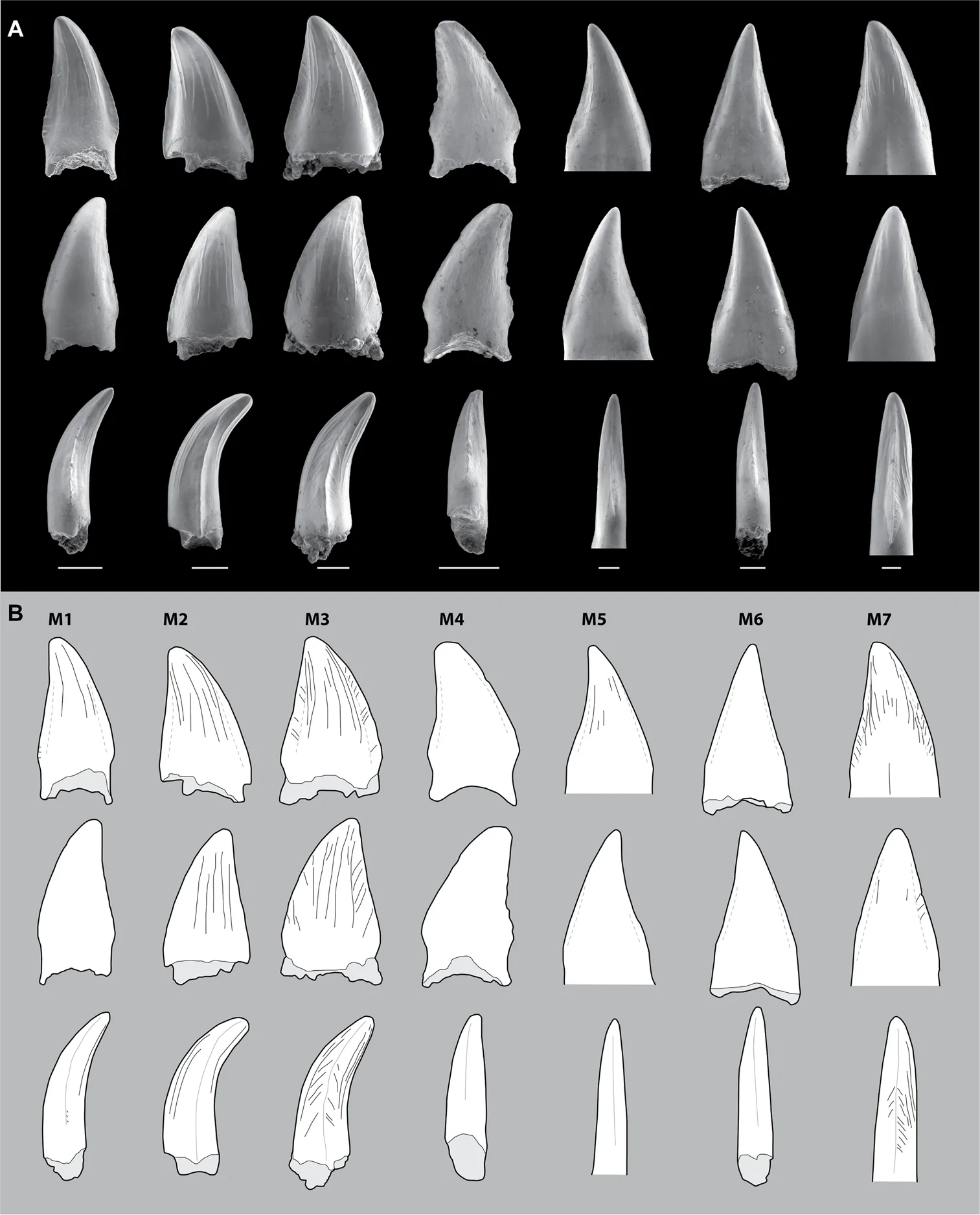
(A) Tooth morphotypes 1–7 to scale with one another in lingual, labial and distal views (top to bottom). (B) Interpretive line drawings of tooth morphotypes. Specimen numbers from left to right: UAMES 52743, UAMES 51585, UAMES 51579, UAMES 52550, UAMES 51524, UAMES 51542 and UAMES 52693. All scale bars represent 500 µm.

**Figure 3.**
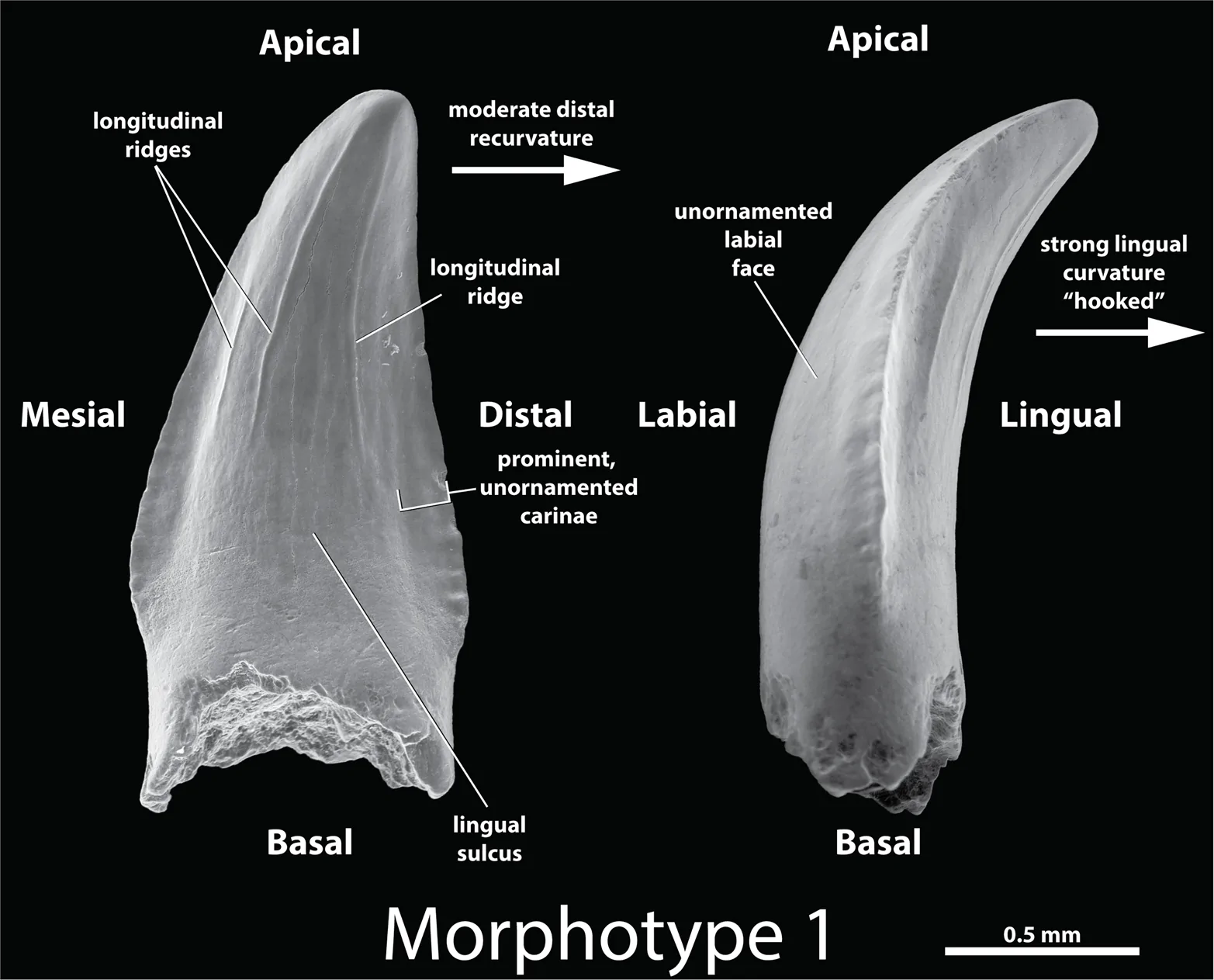
Tooth morphotype 1 annotated to highlight typical morphologies (UAMES 52743).

**Figure 4.**
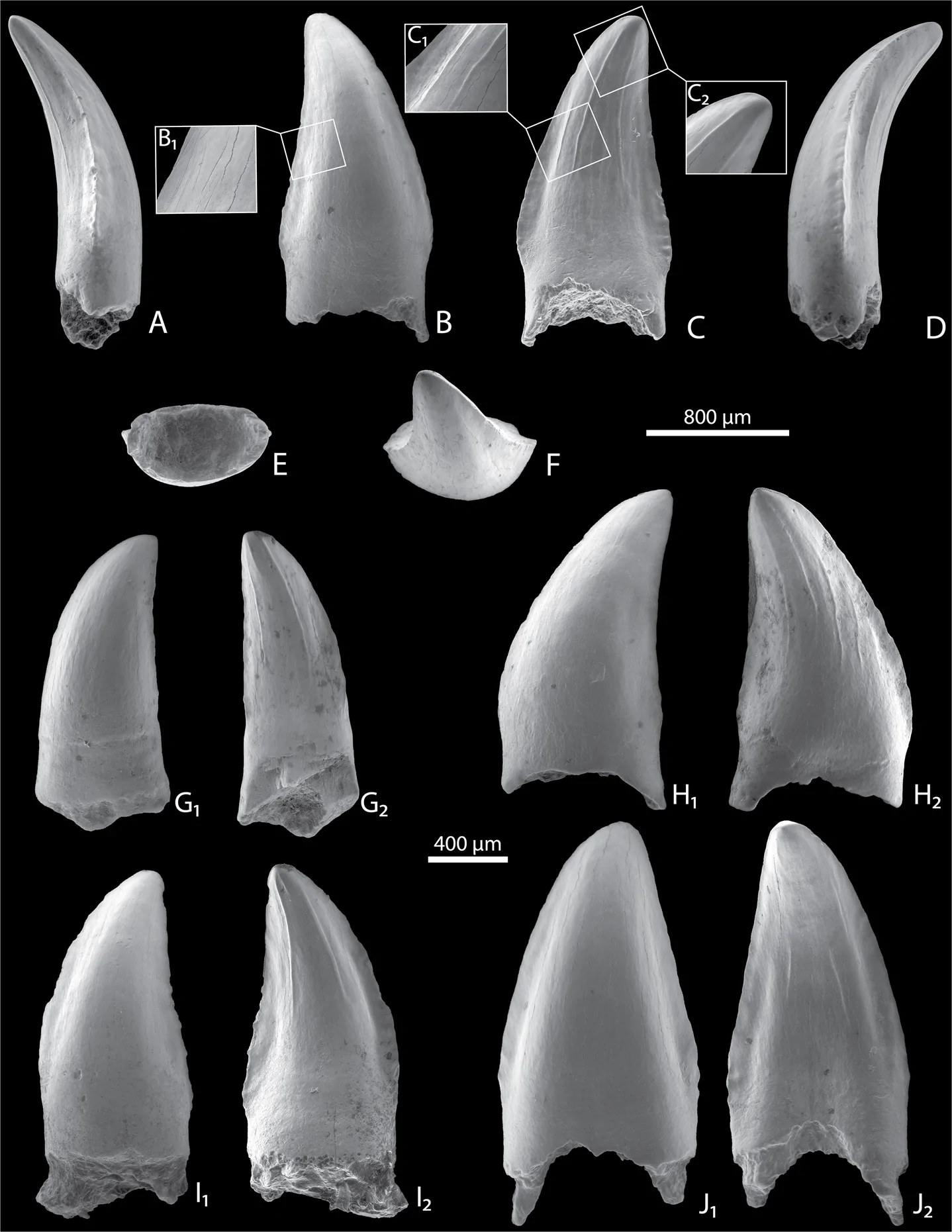
Representative teeth of morphotype 1. (A–F) UAMES 52743 in (A) distal, (B) labial, (C) lingual, (D) mesial, (E) basal and (F) apical view. (B_1_) Close-up of the labial face and distal carina showing unornamented enamel surface. (C_1_) Close-up of the lingual surface showing longitudinal ridges. (C_2_) Close-up of the lingual surface of the apex showing longitudinal ridges. (G) UAMES 51526 in (G_1_) labial and (G_2_) lingual views. (H) UAMES 52465 in (H_1_) labial and (H_2_) lingual views. (I) UAMES 52140 in (I_1_) labial and (I_2_) lingual view. (J) UAMES 52564 in (J_1_) labial and (J_2_) lingual views. The 800 µm scale bar applies to (A–F). The 400 µm scale bar applies to (G–J).

**Figure 5.**
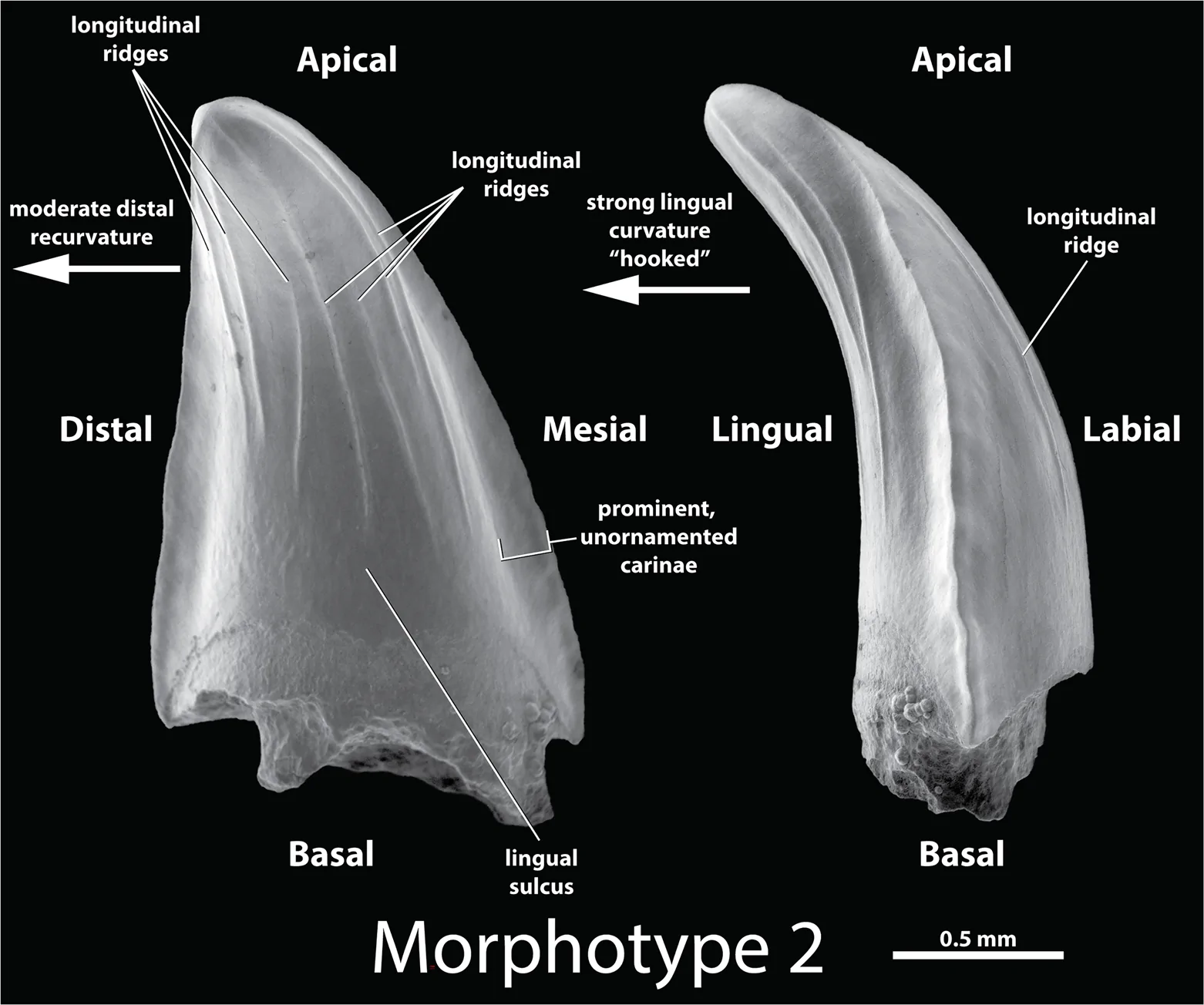
Tooth morphotype 2 annotated to highlight typical morphologies (UAMES 51585). Left, lingual view; right, mesial view.

**Figure 6.**
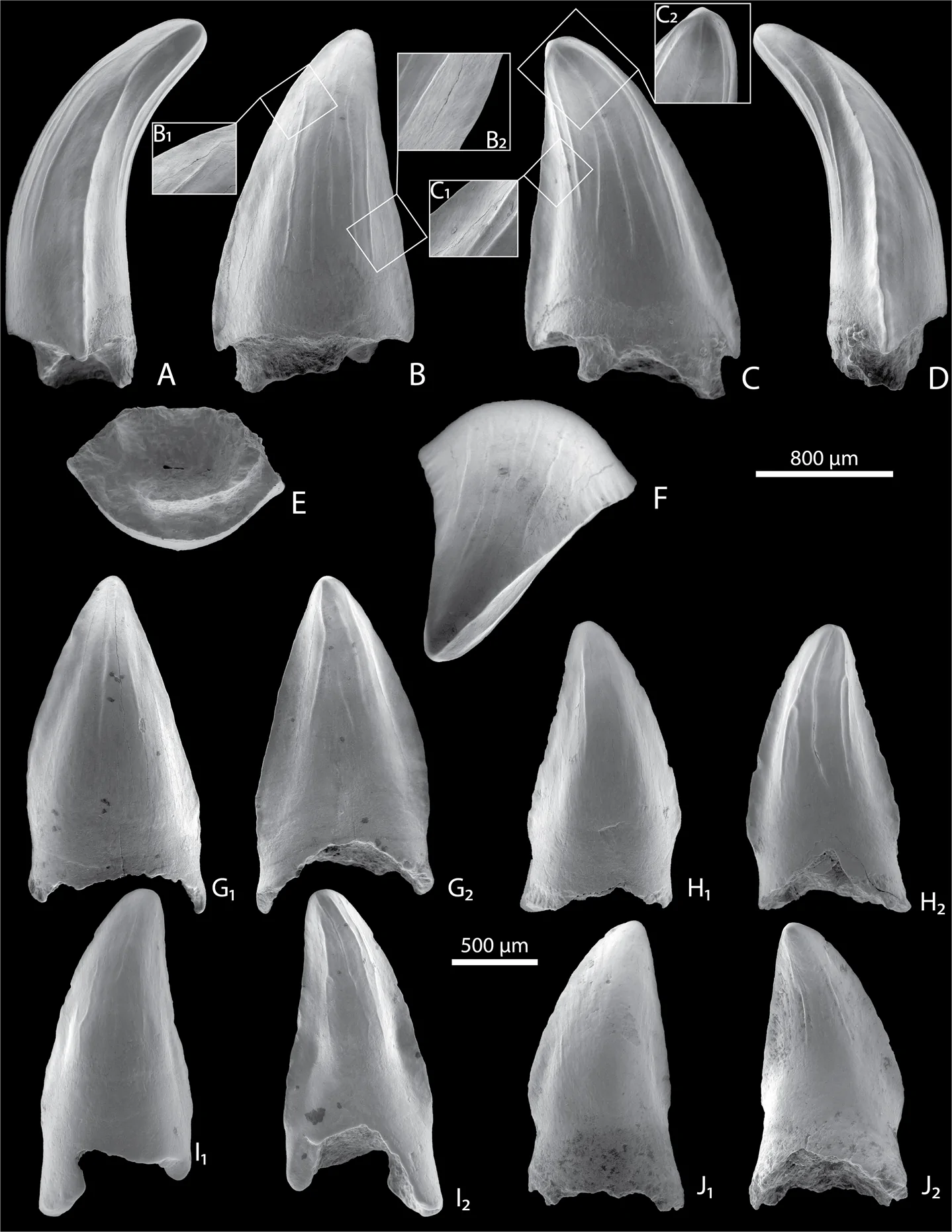
Representative teeth of morphotype 2. (A–F) UAMES 51585 in (A) distal, (B) labial, (C) lingual, (D) mesial, (E) basal and (F) apical view. (B_1_–B_2_) Close-up of the labial face showing longitudinal ridges. (C_1_–C_2_) Close-ups of the lingual surface showing longitudinal ridges. (G) UAMES 51583 in (G_1_) labial and (G_2_) lingual views. (H) UAMES 52677 in (H_1_) labial and (H_2_) lingual views. (I) UAMES 52562 in (I_1_) labial and (I_2_) lingual views. (J) UAMES 52112 in (J_1_) labial and (J_2_) lingual views. The 800 µm scale bar applies to (A–F). The 500 µm scale bar applies to (G–J).

**Figure 7.**
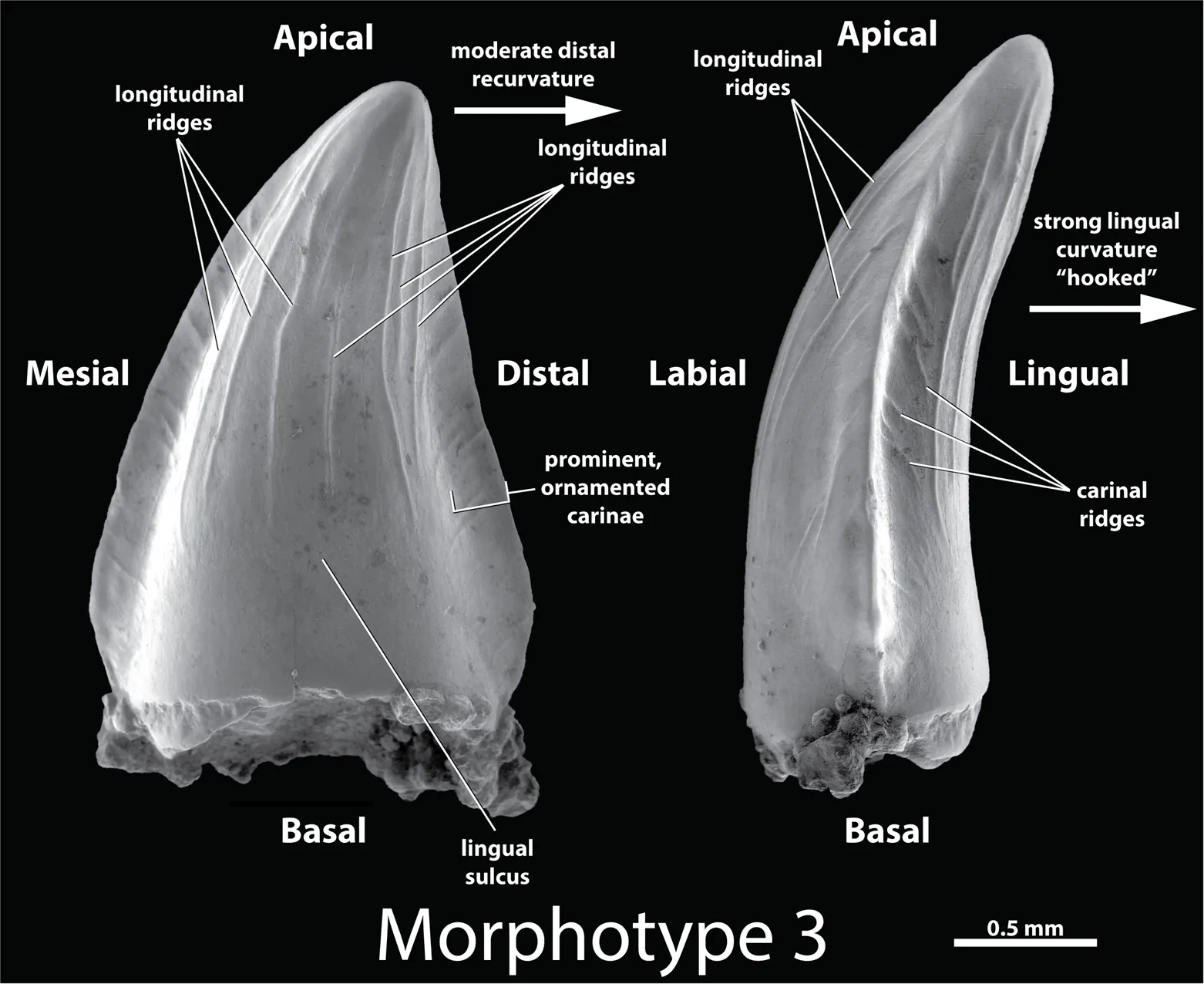
Tooth morphotype 3 annotated to highlight typical morphologies (UAMES 51579). Left, lingual view; right, mesial view.

**Figure 8.**
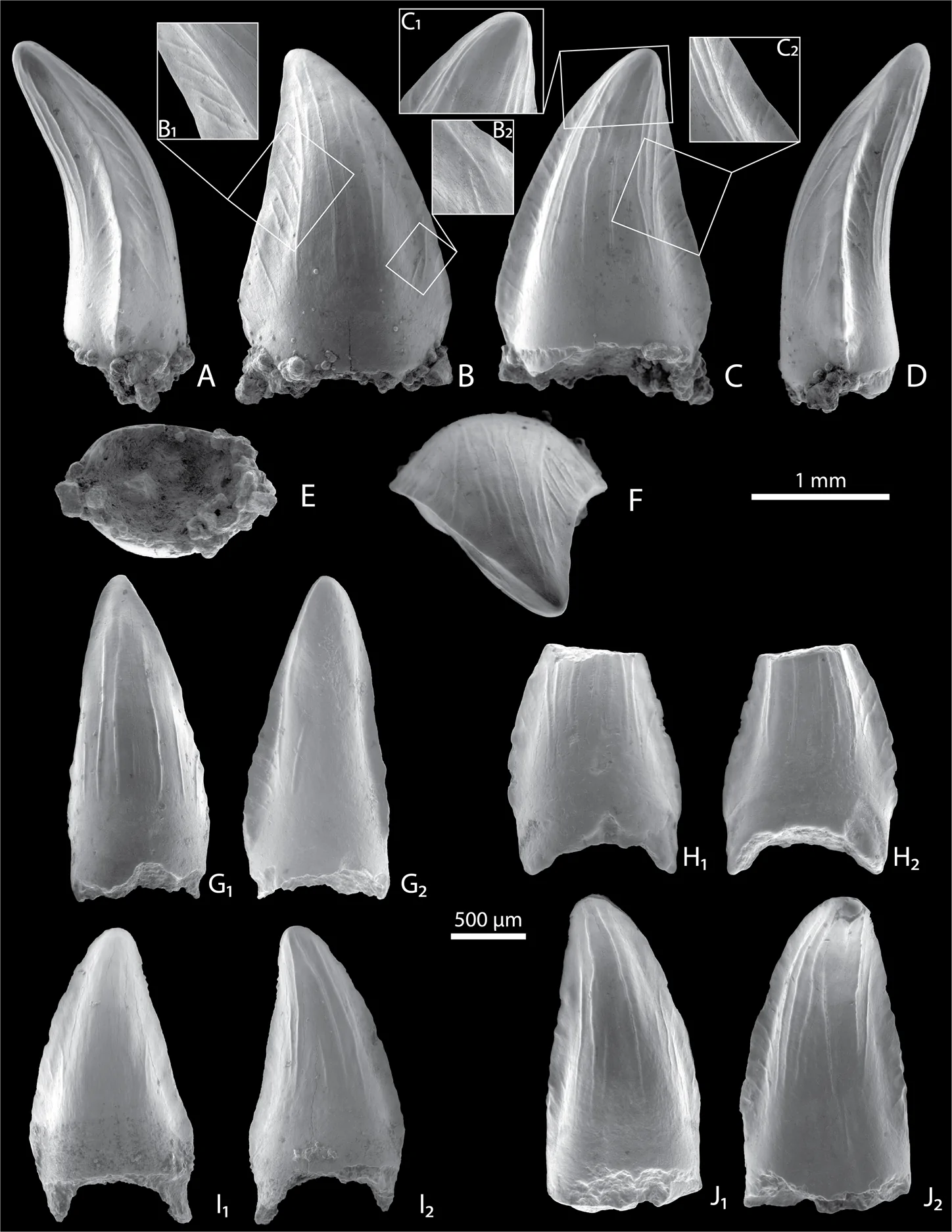
Representative teeth of morphotype 3. (A–F) UAMES 51579 in (A) distal, (B) labial, (C) lingual, (D) mesial, (E) basal and (F) apical view. (B_1_–B_2_) Close-up of the labial face showing longitudinal ridges on the crown and carina. (C_1_–C_2_) Close-ups of the lingual surface showing longitudinal ridges on the crown and carina. (G) UAMES 51528 in (G_1_) labial and (G_2_) lingual views. (H) UAMES 51582 in (H_1_) labial and (H_2_) lingual views. (I) UAMES 52138 in (I_1_) labial and (I_2_) lingual views. (J) UAMES 52565 in (J_1_) labial and (J_2_) lingual views. The 1 mm scale bar applies to (A–F). The 500 µm scale bar applies to (G–J).

**Figure 9.**
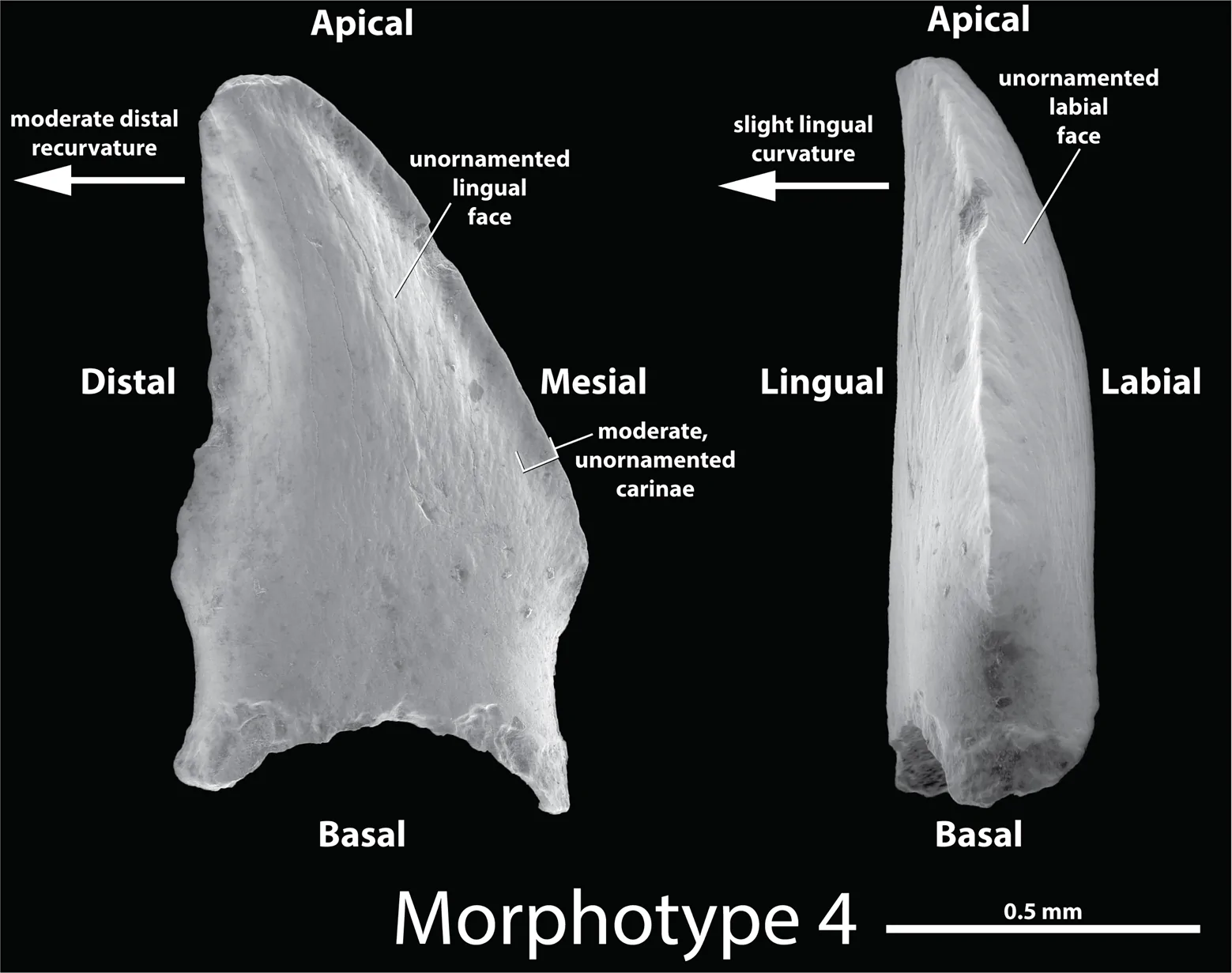
Tooth morphotype 4 annotated to highlight typical morphologies (UAMES 52550). Left, lingual view; right, mesial view.

**Table 3.** Number of avialan teeth of each morphotype in the PCF.

number of teeth of each morphotype									
morphotype	1	2	3	4	5	6	7	damaged (cannot assess)	total number
**no. of teeth**	17	16	9	12	18	10	7	16	105

As the exact sources of variation in avialan tooth morphology are not yet clear, we emphasize the need to not interpret these morphotypes too rigidly, but rather we advocate their use to broadly categorize morphological variation. All seven of the PCF morphotypes exhibit some amount of variation (e.g. precise degree of recurvature, exact number and placement of longitudinal ridges), but until teeth are recovered within jaws, we choose to use broader morphotypes based on general combinations of characters, rather than very narrow categories with precise and strict criteria. Additionally, of all the characters described, we assign the most weight to the presence of enamel ornamentation in different regions of the tooth (labial face, lingual face and/or carinae). Observed avialan teeth in jaws do show variation in carinae width, degree of curvature and recurvature, and overall shape/proportions; however, they do not appear to show significant variation in enamel ornamentation [12,42]. Therefore, we chose to introduce more variation into existing morphotypes rather than erecting morphotypes based on single specimens that vary in only one character.

We further note that we have made an intentional choice to exclude morphometric analyses from this study. There are a handful of issues that arise with morphometrics in this type of sample. As noted by Mohr *et al.* [23], who applied morphometric analyses to fossil bird and crocodyliform teeth, morphometrics are not informative for distinguishing avialan teeth into morphotypes or from the teeth of crocodyliforms. While Mohr *et al.* [23] used linear morphometrics (10 variables and 64 teeth) to visualize (principal components analysis [PCA] ordination) the overall shape of the teeth relative to known avialan teeth, known crocodyliform teeth and non-avialan theropod teeth, it is important to note that the ordination did not allow for quantitative separation of the teeth into their morphotypes. They did this qualitatively, as is done here, based on features such as ‘overall shape in lateral and basal views, curvature, features of the enamel surface and presence or absence of denticles on the mesial and/or distal carinae’ (page 3 in ref. [23]).

Traditional measurement-based morphometrics, and even 2D geometric morphometrics, are only able to analyse physical shape variation in a single orientation and would entirely miss many detailed features like the enamel ornamentation. Very little of the variation in our morphotypes relates solely to the overall shape (silhouette) of the tooth. Instead, most of the variation occurs in the enamel ornamentation on the surfaces of the teeth (e.g. this variation is the only major difference between morphotypes 1, 2 and 3). Even 3D geometric morphometric methods would probably be unable to account for this variation in enamel ornamentation.

It is also important to recognize that even if any landmark-based 2D or 3D morphometrics could capture all fine structures, it would assume homology of the longitudinal ridges, which is not reasonably applicable here given variation in the precise number and position of these ridges from tooth-to-tooth. Even approaches such as pseudolandmarking, which evenly distributes landmarks across the surface of a 3D mesh or 2D curve without implying homology, would be unlikely to resolve delicate enamel structures. While this may be an interesting avenue of future study, a comparative 3D tooth dataset is currently lacking amongst the non-avialan taxa of interest. Given that ecological selective pressures very often shape structures that are morphologically analogous, many crocodyliform teeth *do* resemble avialan teeth. This is why it has been challenging and controversial to distinguish avialan teeth from those of crocodyliforms for two decades. PCA is therefore unlikely to be capable of consistently distinguishing avialan tooth morphotypes from one another or from the teeth of crocodyliforms or theropods. This is precisely why an ecosystem lacking ectotherms, like the PCF, provides a unique opportunity to address this long-standing question.

### 3.1. Morphotype 1

Teeth of morphotype 1 (figures 3 and 4) show strong lingual curvature, giving them a hooked appearance in mesial and distal views, as well as moderate distal recurvature. The lingual surface is indented by a very shallow longitudinal sulcus that runs nearly the length of the crown, shallowing and narrowing apically. The lingual face is ornamented with typically two, but as many as four, longitudinal ridges which originate approximately one-third of the way towards the crown and terminate at the apex. These ridges are typically straight but occasionally are undulatory. The labial face is strongly convex and unornamented. Teeth of this morphotype have prominent and continuous mesial and distal carinae which do not bear denticles, nor are they ornamented with ridges. In sufficiently complete specimens, the crown exhibits a basal constriction and has a relatively mesiodistally expanded root. Exemplar teeth for morphotype 1 include UAMES 52743, 51526, 52465, 52140 and 52564. Measured teeth of this morphotype exhibit a size range of 956–1409 µm for total crown height and 532–876 µm for total basal width.

### 3.2. Morphotype 2

As with morphotype 1, teeth of morphotype 2 (figures 5 and 6) show strong lingual curvature, giving them a hooked appearance in mesial and distal views, as well as moderate distal recurvature. The lingual face is ornamented with longitudinal ridges, though they are typically more numerous than in teeth of morphotype 1. These ridges are typically straight but occasionally are undulatory or branching. As in morphotype 1, the lingual surface is indented by a very shallow longitudinal sulcus that runs the length of the crown, shallowing and narrowing apically. These teeth also have prominent carinae that are not ornamented with ridges. Teeth of morphotype 2 are most easily distinguished from morphotype 1 in that the labial face is also ornamented by longitudinal ridges, though less so than the lingual face. In sufficiently complete specimens, the crown exhibits a basal constriction. Exemplar teeth of morphotype 2 include UAMES 51585, 51583, 52677, 52562 and 52112. Measured teeth of this morphotype exhibit a size range from 932–1783 µm in total crown height and 689–1185 µm in total basal width.

### 3.3. Morphotype 3

Teeth of morphotype 3 (figures 7 and 8) are most similar in overall appearance to those of morphotype 2. The longitudinal ridges are typically straight, but occasionally undulatory. The labial face is also ornamented by longitudinal ridges, though less so than the lingual face. Teeth of this morphotype have prominent and continuous mesial and distal carinae which do not bear denticles. In sufficiently complete specimens, crowns do not exhibit a basal constriction. Teeth of morphotype 3 are most distinct from morphotypes 1 and 2 in that their carinae are ornamented lingually and labially by fine ridges that run apically from the edge of the crown to the margin of the carinae, oriented diagonally to the main apicobasal tooth axis. Exemplar teeth of morphotype 3 include UAMES 51585, 51583, 52677, 52562 and 52112. Measured teeth of this morphotype exhibit a size range from approximately 1104–2229 µm in total crown height and 896–1386 µm in total basal width.

### 3.4. Morphotype 4

Teeth of morphotype 4 (figures 9 and 10) are moderately distally recurved and minimally lingually curved. The mesial and distal margins are strongly asymmetrical in labial and lingual views, and their surfaces are unornamented. Moderately developed, unornamented mesial and distal carinae are present but do not bear denticles. The mesial margin is strongly convex, whereas the distal margin is straight, giving each tooth a somewhat D-shaped outline in lingual and labial views. Shallow sulci indent the basal half of the lingual and labial surfaces. These teeth exhibit a basal constriction, which is more evident in mesial aspect, and the root is mesiodistally expanded relative to the basal constriction. Exemplar teeth of morphotype 4 include UAMES 52550, 52614, 52410, 52403 and 53154. Measured teeth of this morphotype exhibit a size range from approximately 566–1328 µm in total crown height and 394–861 µm in total basal width.

**Figure 10.**
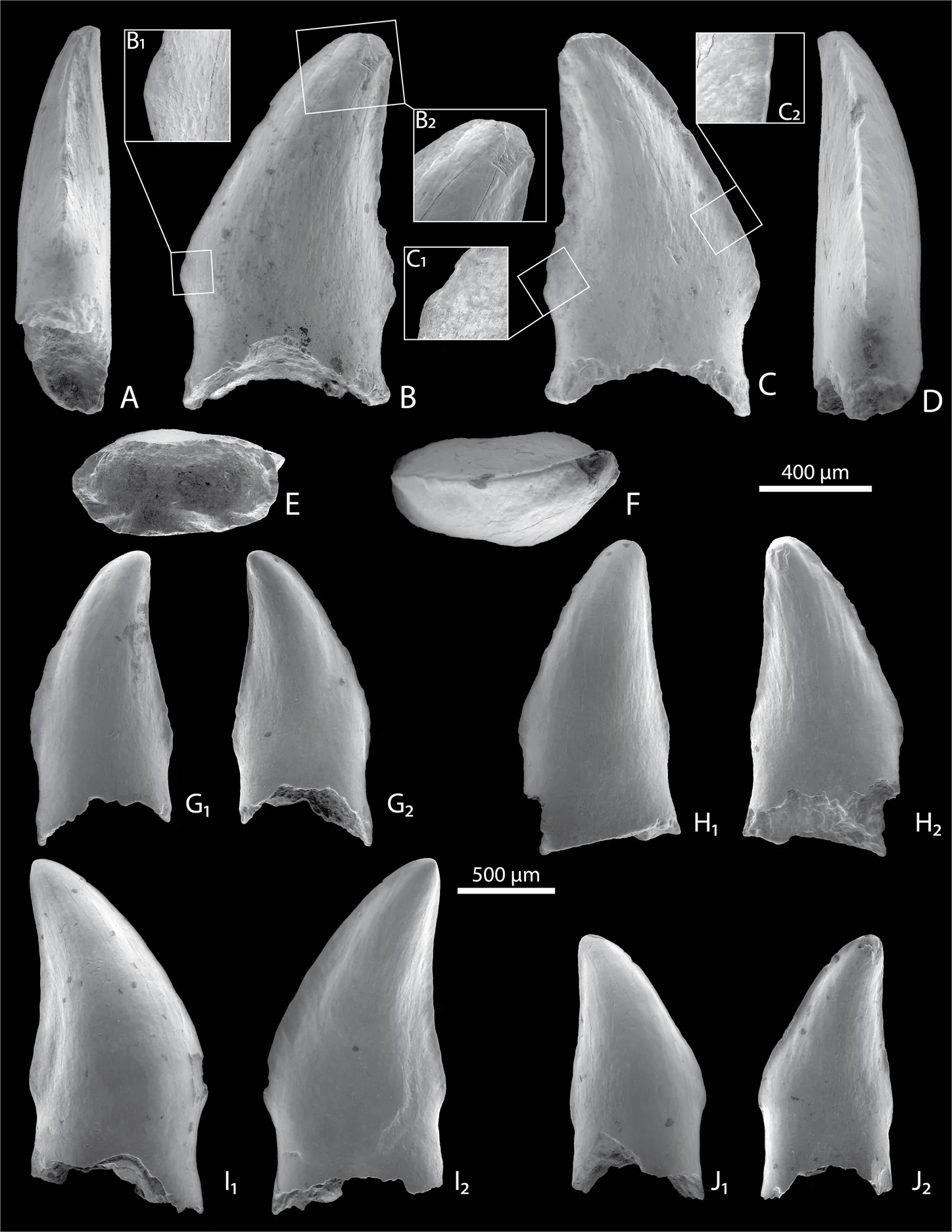
Representative teeth of morphotype 4. (A–F) UAMES 52550 in (A) distal, (B) labial, (C) lingual, (D) mesial, (E) basal and (F) apical view. (B_1_–B_2_) Close-up of the labial face showing unornamented crown and carina. (C_1_–C_2_) Close-ups of the lingual surface showing unornamented crown and carina. (G) UAMES 52614 in (G_1_) labial and (G_2_) lingual views. (H) UAMES 52410 in (H_1_) labial and (H_2_) lingual views. (I) UAMES 52403 in (I_1_) labial and (I_2_) lingual views. (J) UAMES 53154 in (J_1_) labial and (J_2_) lingual views. The 400 µm scale bar applies to (A–F). The 500 µm scale bar applies to (G–J).

### 3.5. Morphotype 5

Teeth of morphotype 5 (figures 11 and 12) are strongly labiolingually compressed, apicobasally tall and slightly to moderately distally recurved. The aspect ratio (apicobasal height to mesiodistal width) is higher than that seen in morphotypes 1–4. In labial and lingual views, the mesial and distal margins are slightly convex and concave, respectively. The teeth bear a strong lingual sulcus which runs longitudinally approximately half the length of the crown. The lingual surface is additionally ornamented by two to four longitudinal ridges which parallel the margins of the sulcus and run the length of the tooth. In some cases, the teeth may bear several shorter parallel longitudinal ridges (figure 12C_1_). The labial surface bears a shallow longitudinal sulcus that shallows and narrows apically but is otherwise unornamented. Mesial and distal carinae are moderately developed, continuous, unornamented and do not bear denticles. In one specimen (UAMES 51577), the carinae are prominent. In sufficiently complete specimens, crowns exhibit a slight basal constriction and exhibit a relatively expanded root. In one specimen that preserves a nearly complete root (UAMES 51524), the root exhibits a narrow and deep replacement tooth pit on its lingual surface (figure 12C). Exemplar teeth of morphotype 5 include UAMES 51524, 52566, 52981, 42721 and 53151. Measured teeth of this morphotype exhibit a size range from approximately 2617–7051 µm in total crown height and 1374–4549 µm in total basal width.

**Figure 11.**
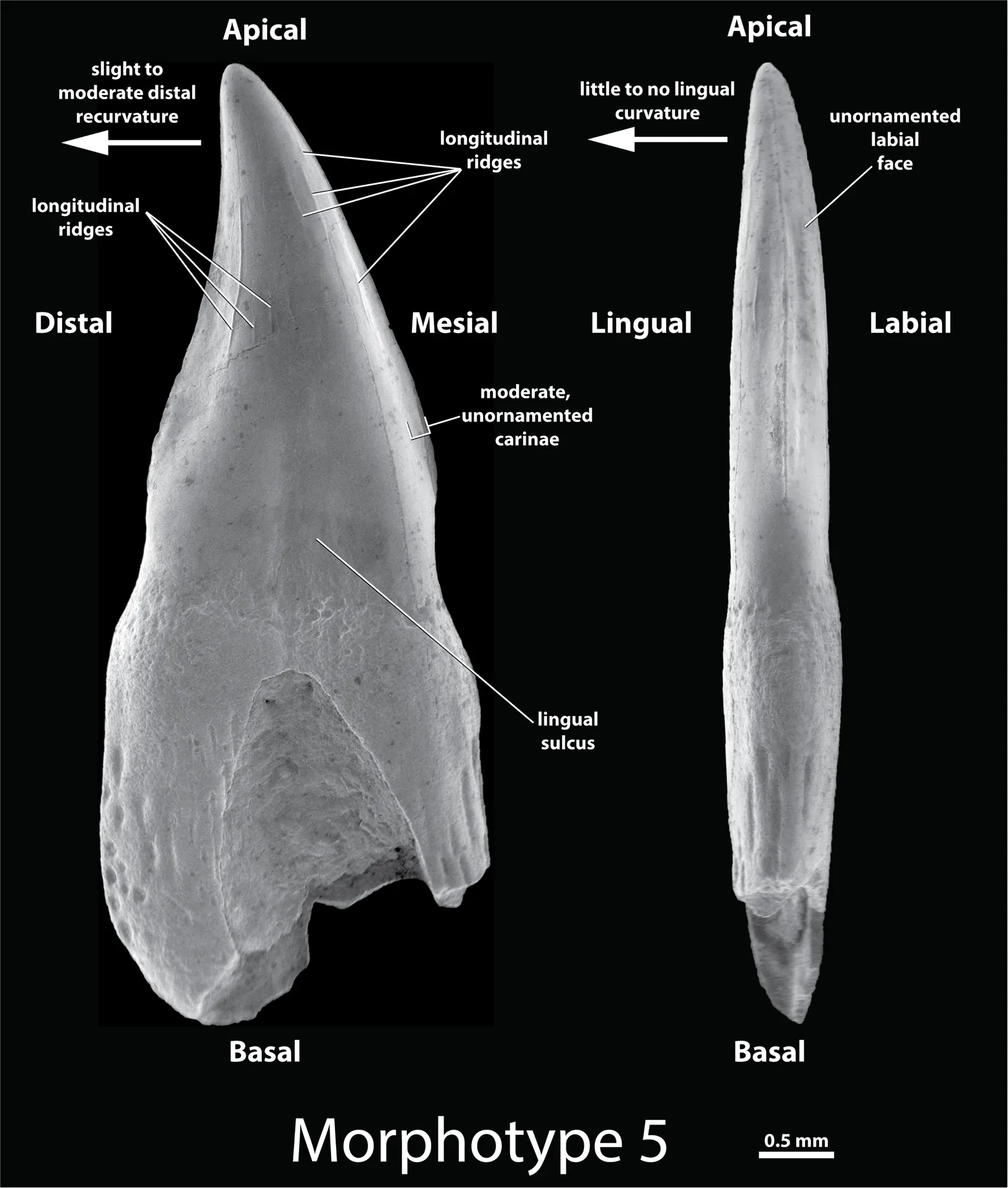
Tooth morphotype 5 annotated to highlight typical morphologies (UAMES 51524). Left, lingual view; right, mesial view.

**Figure 12.**
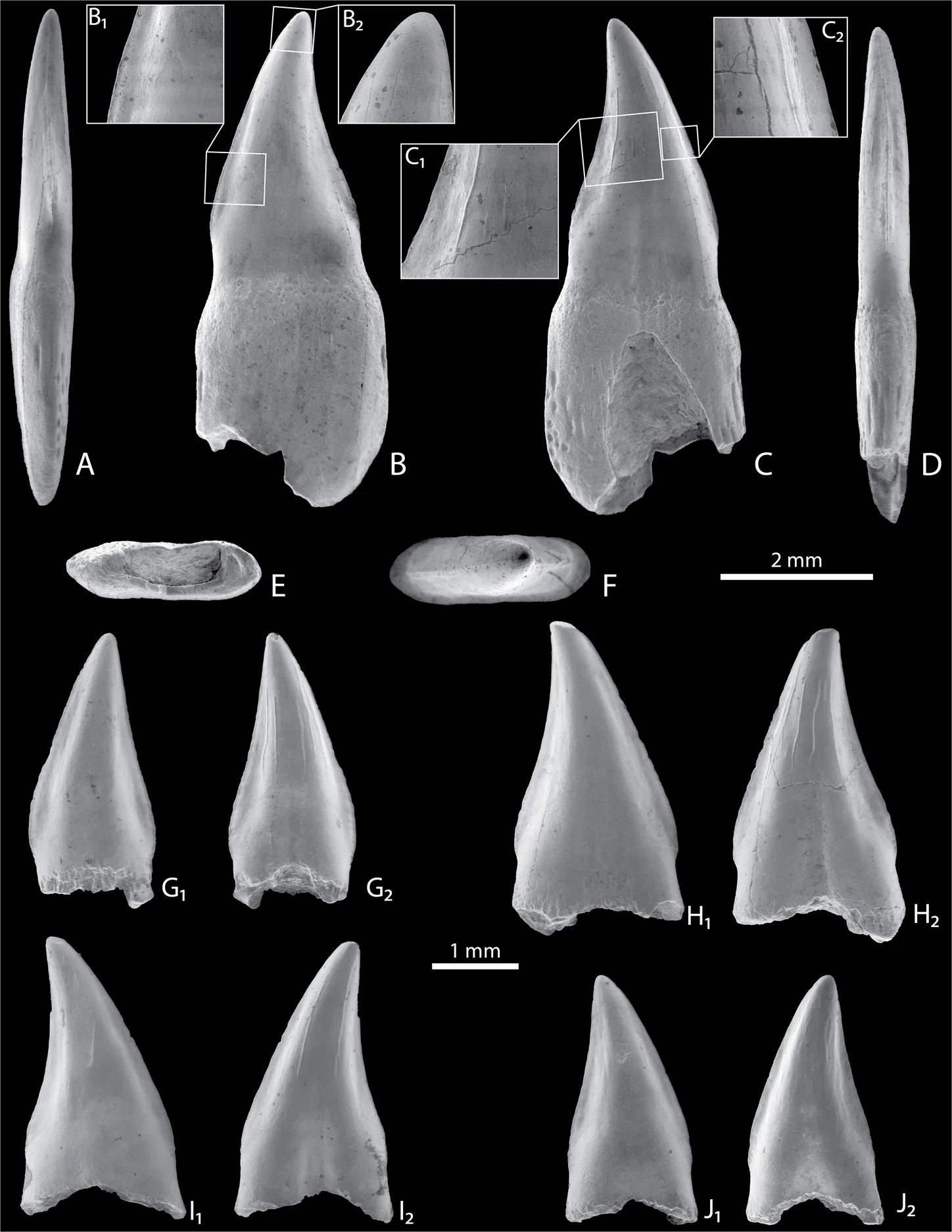
Representative teeth of morphotype 5. (A–F) UAMES 51524 in (A) distal, (B) labial, (C) lingual, (D) mesial, (E) basal and (F) apical view. (B_1_–B_2_) Close-up of the labial face showing unornamented crown and carina. (C_1_–C_2_) Close-ups of the lingual surface longitudinal ridges on the crown and unornamented carina. (G) UAMES 52566 in (G_1_) labial and (G_2_) lingual views. (H) UAMES 52981 in (H_1_) labial and (H_2_) lingual views. (I) UAMES 42721 in (I_1_) labial and (I_2_) lingual views. (J) UAMES 53151 in (J_1_) labial and (J_2_) lingual views. The 2 mm scale bar applies to (A–F). The 1 mm scale bar applies to (G–J).

### 3.6. Morphotype 6

Teeth of morphotype 6 (figures 13 and 14) are nearly isosceles triangular in labial and lingual view and are only very slightly to moderately distally recurved and strongly compressed laterally. The lingual surface bears a deep longitudinal sulcus which nearly reaches the apex. The teeth are smooth and entirely unornamented. Mesial and distal carinae are prominent. They originate at the base of the crown and terminate well before the apex. Teeth of this morphotype do not exhibit a basal constriction. Exemplar teeth of morphotype 6 include UAMES 51542, 52418, 52551, 51580 and 52983. Measured teeth of this morphotype exhibit a size range from approximately 1901–3356 µm in total crown height and 1280–2130 µm in total basal width.

**Figure 13.**
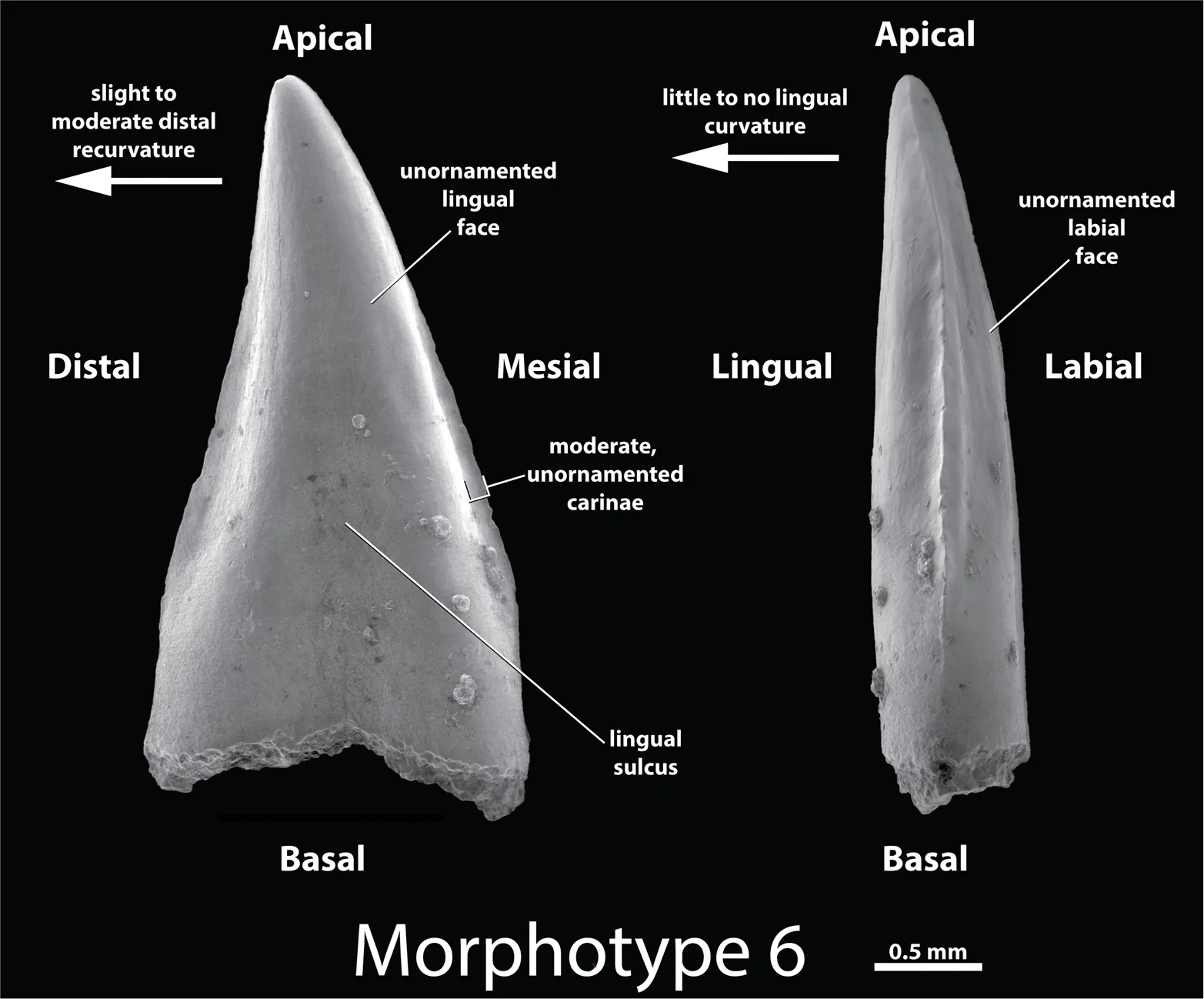
Tooth morphotype 6 annotated to highlight typical morphologies (UAMES 51542). Left, lingual view; right, mesial view.

**Figure 14.**
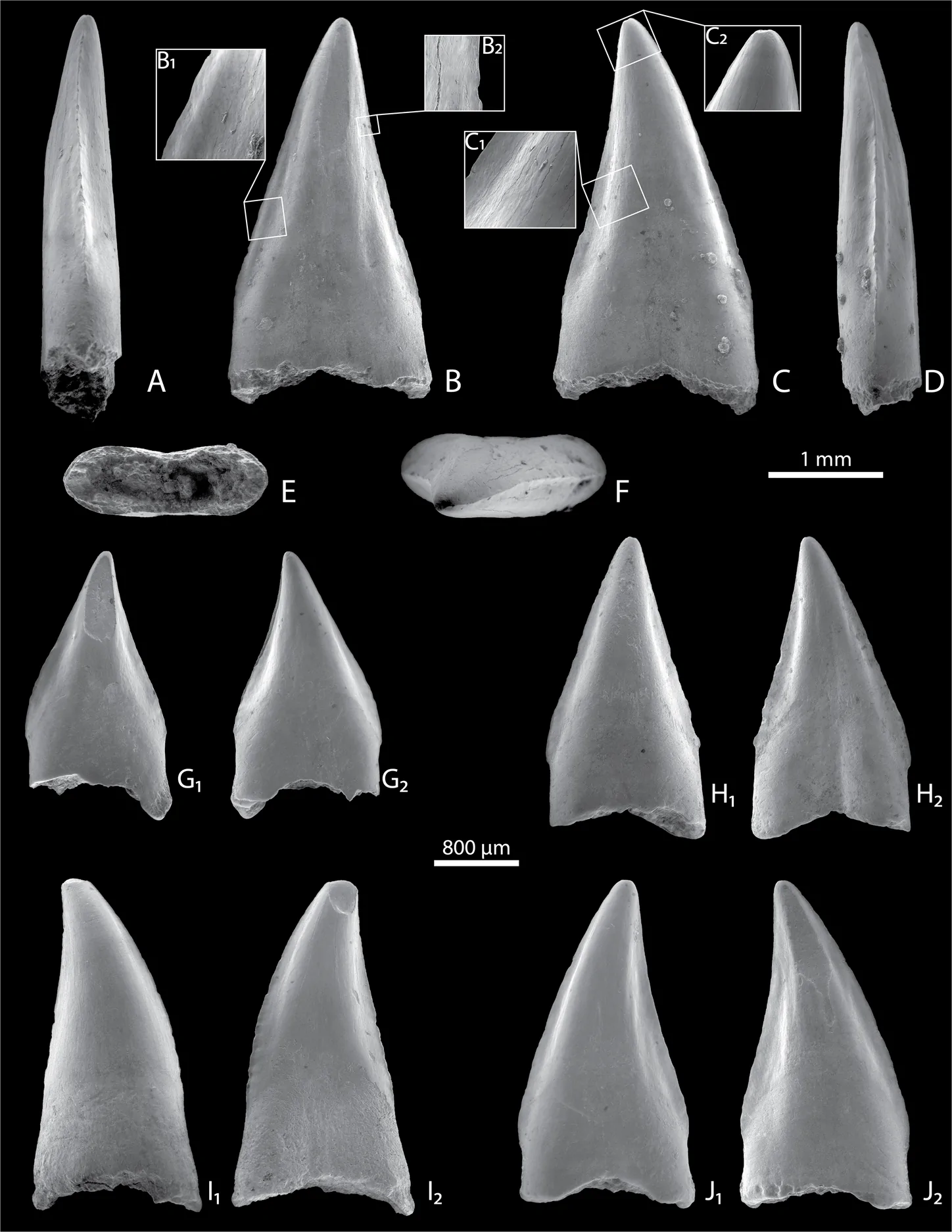
Representative teeth of morphotype 6. (A–F) UAMES 51542 in (A) distal, (B) labial, (C) lingual, (D) mesial, (E) basal and (F) apical view. (B_1_–B_2_) Close-up of the labial face showing unornamented crown and carina. (C_1_–C_2_) Close-ups of the lingual surface showing unornamented crown and carina. (G) UAMES 52418 in (G_1_) labial and (G_2_) lingual views. (H) UAMES 52551 in (H_1_) labial and (H_2_) lingual views. (I) UAMES 51580 in (I_1_) labial and (I_2_) lingual views. (J) UAMES 52983 in (J_1_) labial and (J_2_) lingual views. The 1 mm scale bar applies to (A–F). The 800 µm scale bar applies to (G–J).

### 3.7. Morphotype 7

Teeth belonging to morphotype 7 (figures 15 and 16) are slightly to strongly distally recurved and exhibit very little to no lingual curvature. They bear a shallow lingual sulcus which shallows apically. Multiple prominent, parallel longitudinal ridges run nearly the length of the crown on the lingual and labial faces. The labial face is typically more heavily ornamented than the lingual face. Continuous mesial and distal carinae are ornamented with irregular striations that run apically from the edge of the crown to the margin of the carinae, oriented diagonally to the main apicobasal tooth axis. The enamel surfaces, on both the lingual and labial faces, exhibit an arch which bows apically from the base of the carinae, basal to which dentine is exposed. The teeth do not exhibit a basal constriction, nor an expanded root. Exemplar teeth of morphotype 7 include UAMES 52693, 52266, 52142, 52590 and 41654. Measured teeth of this morphotype exhibit a size range from 2123 to 5827 µm in total crown height and 1397–3564 µm in total basal width.

**Figure 15.**
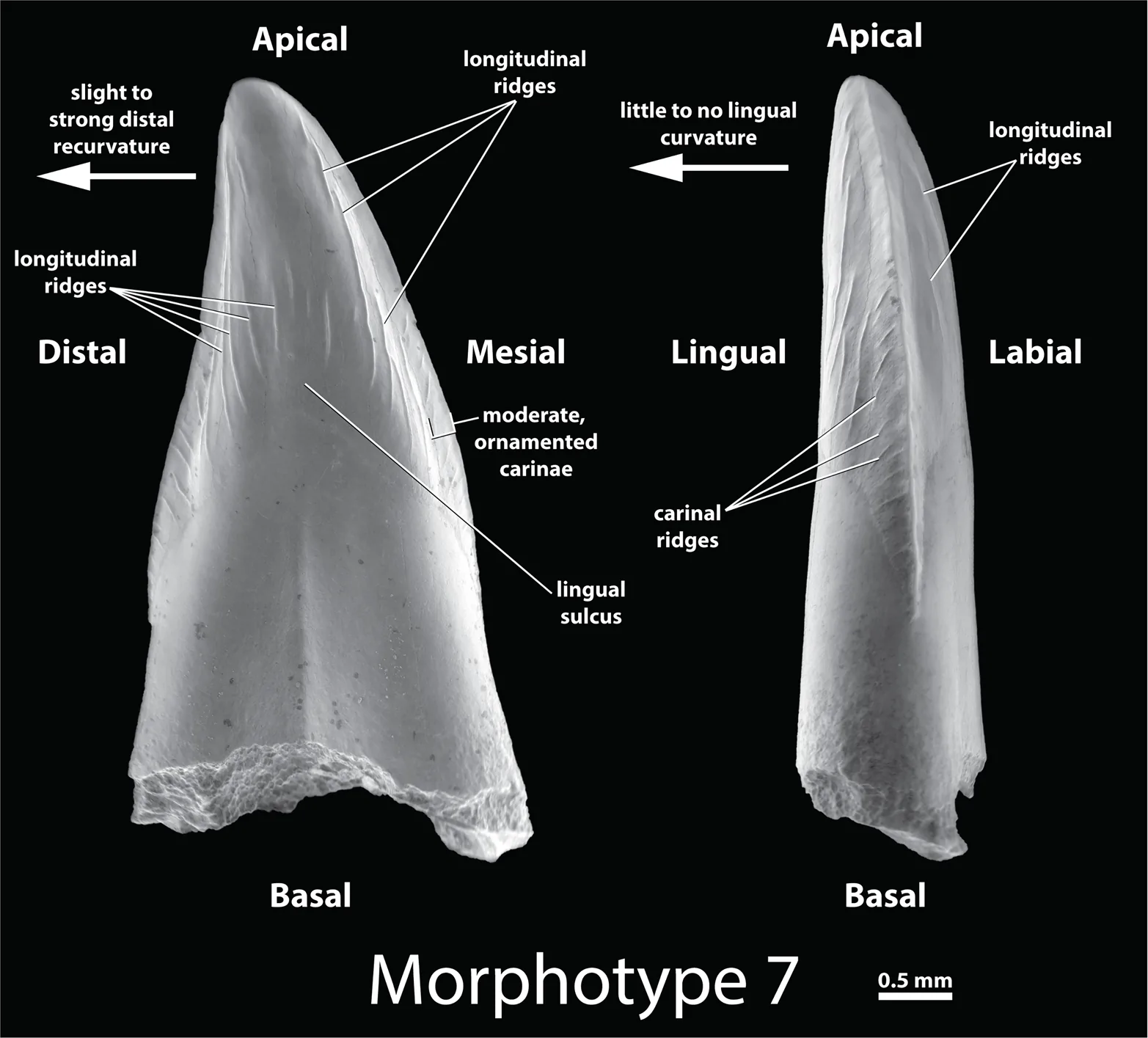
Tooth morphotype 7 annotated to highlight typical morphologies (UAMES 52693). Left, lingual view; right, mesial view.

**Figure 16.**
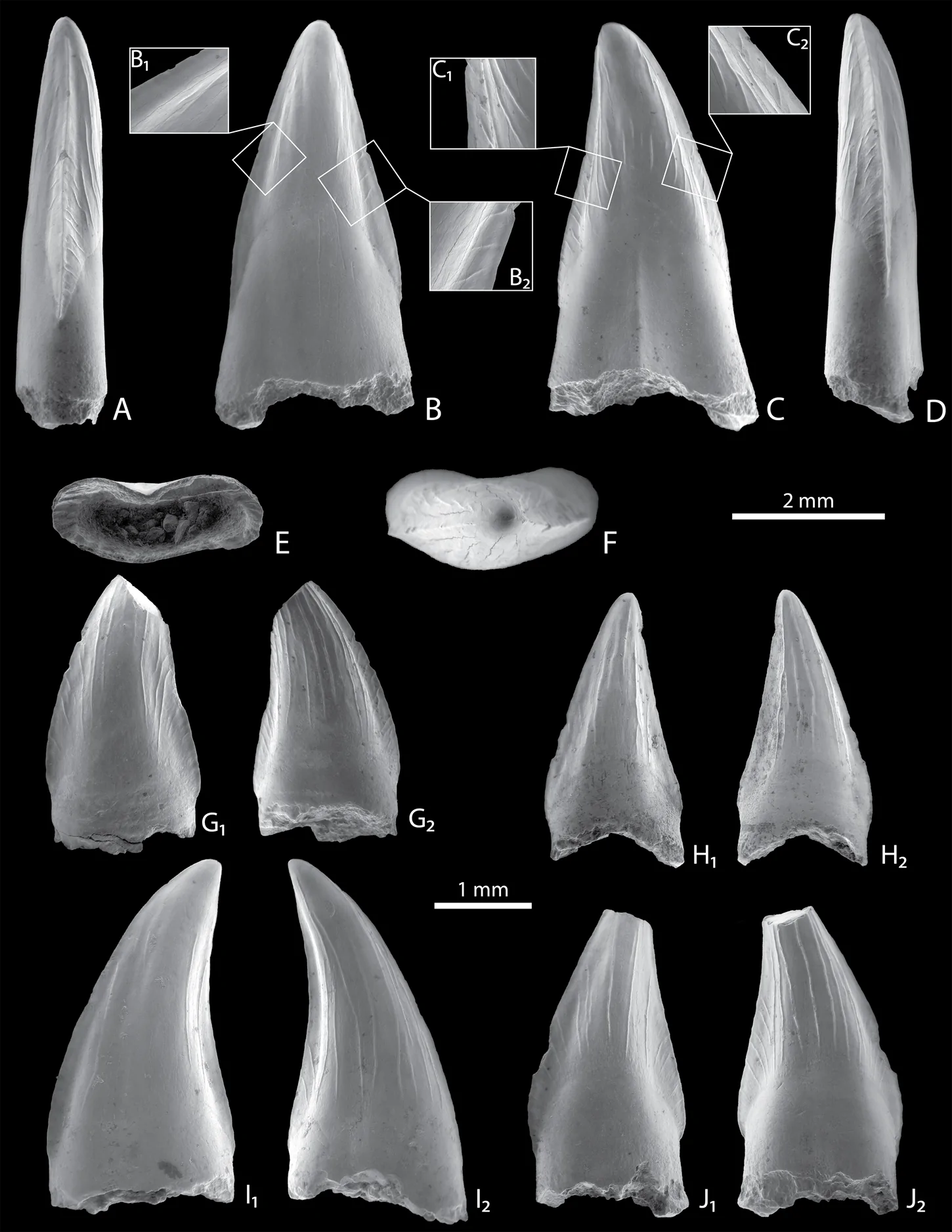
Representative teeth of morphotype 7. (A–F) UAMES 52693 in (A) distal, (B) labial, (C) lingual, (D) mesial, (E) basal and (F) apical view. (B_1_–B_2_) Close-ups of the labial face showing longitudinal ridges on the crown and carina. (C_1_–C_2_) Close-ups of the lingual surface showing longitudinal ridges on the crown and carina. (G) UAMES 52266 in (G_1_) labial and (G_2_) lingual views. (H) UAMES 52142 in (H_1_) labial and (H_2_) lingual views. (I) UAMES 52590 in (I_1_) labial and (I_2_) lingual views. (J) UAMES 41654 in (J_1_) labial and (J_2_) lingual views. The 2 mm scale bar applies to (A–F). The 1 mm scale bar applies to (G–J).

## 4. Discussion

### 4.1. Comparison of the PCF teeth with non-avialan theropods

Differences between ‘Aves indet.’ teeth and those of non-avialan theropods have been evident since their first description in 2001 [13]. They were originally described as differing from the teeth of all other known theropods in the following features: (i) small; (ii) stout but strongly laterally compressed; (iii) crown slightly recurved; (iv) carinae on both the anterior (mesial) and posterior (distal) edge; (v) lacking denticles; and (vi) constricted at the base of the crown. Sankey *et al.* [14] reiterated many of these features, additionally contributing the following character: (vii) sulci on their labial and lingual faces. While many of these features are present in non-avialan theropod teeth, the unique combination of characters has allowed for putative avialan teeth to be distinguished from other tooth morphologies for over two decades [13,14,22,23].

One of the most obviously avialan morphologies is the lack of denticles, which are otherwise present in most non-avialan theropods. While the occasional isolated denticle-bearing tooth has been identified as avialan [14,16], there are no denticle-bearing teeth known from avialan jaws (see §4.8). In fact, Mohr *et al.* [23] point out that these denticle-bearing teeth do bear a resemblance to the teeth of non-avialan theropods and even some crocodyliforms (e.g. *Doratodon* [43]). Dumont *et al.* [12] further suggest that all putative avialan teeth with denticles are most likely from non-avialan theropods. Given that there are abundant small non-avialan theropods, and even crocodyliforms, in the Late Cretaceous that are known to have denticle-bearing teeth, we echo Dumont *et al.*'s [12] suggestion that these rare denticle-bearing putative avialan teeth are most likely not avialan.

The PCF preserves comprehensive samples of teeth, ranging from perinatal to adult, for most of the formation's non-avialan theropods [27]. Most of the PCF theropods can be ruled out on the basis of size alone, with most adult non-avialan theropod teeth far exceeding the size of the avialan teeth and with the largest avialan tooth possessing a crown height of only 4 mm. A tyrannosaur identity can be easily ruled out on the basis of morphology alone. Furthermore, even the smallest young-of-the-year tyrannosaur teeth possess a crown height of 8.4 mm [27], far exceeding the size of the largest PCF avialan teeth. Additional theropods known from the PCF include at least one troodontid, two dromaeosaurids, *Paronychodon* and *Richardoestesia* (cf. *R. isosceles*) [27]. Most adult troodontid teeth from the PCF are notably larger than those from other Late Cretaceous formations, measuring 9.78 mm on average (versus 4.96 mm in Alberta and Montana) [44], far exceeding the size of the avialan teeth. Further, all known troodontid teeth from the PCF bear denticles, including a perinatal specimen with a crown height of only 1.9 mm (19.4% adult size) [27]. *Richardoestesia* can similarly be ruled out as a possible identity as the teeth of this taxon always bear denticles [15,23].

Although the teeth of some troodontids (e.g. *Urbacodon* [45] and *Byronosaurus* [46]) are known to lack denticles, no adult-sized troodontid teeth entirely lacking denticles are known from the PCF. This absence is unlikely to be related to lack of sampling, as the UAMES collection currently houses a collection of >100 adult-sized troodontid teeth from the PCF, all of which bear denticles in various combinations (e.g. no mesial denticles but large distal denticles; small but pronounced hook-like mesial and distal denticles).

Both dromaeosaurids in the PCF are known from adult material, including denticle-bearing teeth. Saurornitholestine teeth from the PCF are large, measuring approximately 10.1 mm, which is comparable to *Saurornitholestes langstoni* from Alberta (crown height = 9.17 mm) [27]. The saurornitholestine is further known from a tooth that, despite being from a perinatal or neonatal individual, still bears denticles on both carinae [27].

The only non-avialan theropod teeth from the PCF lacking denticles belong to *Paronychodon* [17], a genus based solely on teeth whose taxonomic affinities remain uncertain, though some may represent certain premaxillary teeth of dromaeosaurids like *Saurornitholestes* [47]. The adult morphology of *Paronychodon* teeth is not consistent with the combination of features seen in the avialan morphotypes described here, differing markedly in crown shape, basal tooth cross-sectional shape and longitudinal ridge morphology, among other features [14]. For example, the longitudinal ridges of *Paronychodon* teeth are typically broader and more rounded than the very sharp, thin ridges of the avialan teeth described here. We consider it less likely that the small tooth morphotypes described here belong to a hypothetical perinatal *Paronychodon*, as this would require major ontogenetic change in tooth morphology. There is no evidence for such a change in this taxon, and more broadly very little evidence for such changes in non-avialan theropods in general. The one potential previously proposed exception involving major ontogenetic tooth shape change is the immature megalosaurid *Sciurumimus*, but it is important to note that this taxon is not known from adult specimens, so it cannot be said definitely how their teeth change through ontogeny [48]. In total, we consider it unlikely that the teeth described here belong to any of the non-avialan theropods known from the PCF.

The only other theropods that bear some overall similarity to those of putative avialan teeth are those of noasaurids, which sometimes share similarities, including twisting of the carina along the tooth's curvature [49]. However, this clade is currently only known from Gondwana and China, and there is no evidence of their presence in any well-sampled North American formations. Additionally, there are enough morphological differences to distinguish them, with the teeth of *Masiakasaurus* bearing prominent denticles and fluting on the lingual face, rather than the sharp, thin longitudinal ridges seen in avialan teeth [49]. Further, while *Limusaurus* does have unserrated teeth, the crowns are apicobasally much taller and mesiodistally narrower, giving them a ‘tall and thin’ appearance [50]. In contrast, the PCF avialan teeth are triangular, with mesiodistally broad bases that taper towards the apex. Further, the teeth of *Limusaurus* are very straight at the base with parallel mesial and distal edges for most of the height and are abruptly recurved near the apex, rather than smoothly recurved along their entire height as in the avialan teeth.

### 4.2. Differentiation of the PCF teeth from ornithischians

Some avialan teeth have occasionally been noted to superficially resemble the premaxillary teeth of some ornithischians [22,23], including thescelosaurids and pachycephalosaurids, both of which occur in the PCF [51]. While there are some superficial similarities between certain ornithischian premaxillary teeth (e.g. *Thescelosaurus* [51]) and certain avialan teeth, they can be easily distinguished.

The most similar ornithischian teeth in overall profile to avialan teeth are the labiolingually compressed premaxillary teeth of pachycephalosaurs (e.g. *Stegoceras* [52]). However, they are still easily distinguishable as the teeth of pachycephalosaurs bear denticles [52].

Even in those ornithischian premaxillary teeth which lack denticles, such as those of some thescelosaurids [52], the tooth crowns are considerably more bulbous and conical, a morphology often described as ‘turnip-shaped’ [51]. This is especially discernable in mesial and distal views of such ornithischian teeth (e.g. *Thescelosaurus* [51,52]), whereas avialan teeth are visibly labiolingually compressed. While the premaxillary tooth crowns of other thescelosaurids such as *Fona* and *Zephyrosaurus* are slightly more labiolingually compressed than those of *Thescelosaurus*, this condition is still far less than that seen in the sample of PCF avialan teeth [53,54]. This more rounded cross section of thescelosaurid teeth is also reflected in the tooth root, with the roots of thescelosaurid teeth being round to slightly oval in cross section [53–55], contrasting the strong labiolingual compression seen in the root of the PCF avialan teeth.

Further, while the teeth of some ornithischians, such as *Thescelosaurus*, do have ridges that run the length of the tooth, they are much broader and more regularly spaced, present along the tooth's entire surface, and are separated by grooves [51,52]. The PCF avialan teeth described here, in contrast, bear much finer, narrower ridges which do not cover the entire surface of the tooth crown and are not separated by grooves. Also, and significantly, the premaxillary teeth of ornithischians either lack wide carinae, such as *Thescelosaurus* [52,55], or, if carinated, generally bear denticles such as *Fona* [53] and do not exhibit the wide, smooth carinae that are present in the PCF avialan teeth.

Finally, likely owing to occlusion with the keratinous gnathotheca of the predentary, the premaxillary teeth of thescelosaurids generally bear distinct wear facets, expressed as either near-vertical facets lingually or blunted apices [51,53,54]. Similar distinct wear facets are entirely absent in the sample of PCF avialan teeth.

There is therefore no evidence to assign any of the PCF avialan teeth described here to an ornithischian identity.

### 4.3. Differentiation of the PCF teeth from crocodyliforms

Morphological similarities between isolated avialan teeth and crocodyliform teeth are well-established [22,23]. The most striking similarity between teeth of avialans and those of crocodyliforms is the complex enamel ornamentation, specifically the longitudinal ridges on the labial and lingual faces. However, there are major distinguishing features that set avialan teeth apart from those of adult crocodyliforms, particularly their vastly smaller size and extreme labiolingual compression [22]. It is worth noting that, while uncommon, some Cretaceous crocodyliforms do have labiolingually compressed, lingually curved and distally recurved teeth (e.g. *Ogresuchus* [56]), though these teeth are much larger and more robust than typical putative avialan teeth; discovery of very small perinatal teeth from individuals of such crocodyliforms is needed to directly compare with avialan teeth.

Putative avialan teeth most closely resemble those of juvenile crocodyliforms. Mohr *et al.* [23] compared putative avialan teeth from the Belly River Group to those of extant juvenile *Alligator* and *Caiman*. They demonstrated that, while most adult crocodyliform teeth are conical, the teeth of juvenile crocodyliforms are labiolingually compressed. Additionally, they conclude that crocodyliform teeth bear complex longitudinal ridges, similar to those seen in isolated putative avialan teeth and unlike those of any avialan known from teeth in jaws (see §4.5). However, while longitudinal ridges are known from crocodyliform teeth and, in fact, are more prominent in the smaller known teeth of the extinct *Leidyosuchus*, these small *Leidyosuchus* teeth are still easily distinguishable from putative avialan teeth. Even the first referral of teeth to ‘Aves indet.’ noted that, while they are somewhat similar, avialan teeth can be distinguished from those of *Leidyosuchus* on the basis of greater lateral compression and stronger distal recurvature [13]. Additionally, while some juvenile crocodyliform teeth are slightly lingually curved (e.g. *Alligator*), none could be reasonably considered ‘hooked’ to the same degree as *Hesperornis* and many of the teeth described herein.

These similarities highlight why, for nearly 20 years, these putative avialan teeth have been extensively compared with the teeth of crocodyliforms to attempt to sort out their true identity. Even Mohr *et al.*'s extensive qualitative and quantitative comparisons to the teeth of extant and extinct crocodyliforms were still not able to unambiguously resolve the taxonomic identity of the teeth [23]. It is because these putative avialan teeth look so similar to crocodyliform teeth that two decades of comparative morphology, and even quantitative analyses, have come no closer to solving this problem. Instead, the only way to truly resolve the identity of these teeth is to investigate an ecosystem that is completely absent of crocodyliforms.

### 4.4. Absence of crocodyliforms in the Prince Creek Formation

Located at a palaeolatitude of 80–85°N [27,57], the PCF was subject to harsh winters in which perennial residents endured approximately 120 days of continuous darkness, freezing conditions (−2 ± 3.9°C cold month mean temperature [CMMT]) and occasional snowfall [27,58], resulting in uninhabitable conditions for thermally restricted ectothermic tetrapods. We place particular significance on the complete absence of terrestrial ectothermic taxa [27,30] in the PCF, including crocodyliforms, turtles, snakes, lizards, choristoderes and lissamphibians, despite extensive sampling at several microvertebrate-bearing localities. Thermophysiological research on extant crocodylians indicate a minimum CMMT tolerance of 5.5°C [59–62]. As Cretaceous crocodyliforms have thermophysiologies that are believed to be comparable to extant crocodylians [26], they would be precluded from the PCF as the CMMT estimates of the palaeoecosystem preserved in the PCF fall well below this threshold (by approx. 7.5°C).

Moreover, modern crocodylians have been found to be even more thermally restricted than turtles and snakes [60], both of which are also entirely absent from the PCF. A decline or absence of thermally restricted ectotherms is also noted in the Horseshoe Canyon Formation of Alberta, Canada, coinciding with a period of decreasing temperatures. Brinkman [63] notes a decline in turtle diversity, and Larson *et al.* [18] note an absence of crocodyliforms in the middle units of the Horseshoe Canyon Formation during the late Campanian and earliest Maastrichtian, corresponding to the same period of cooling which produced high seasonality in the extremely high-latitude PCF. Similarly, while a relatively abundant squamate fauna is known from sites of late Campanian to early Maastrichtian age in the Wapiti Formation of northern Alberta, the low abundance of crocodyliforms in the formation as a whole [20,64] suggests they are near the northern edge of their range. It is notable that at a palaeolatitude of about 58°N [18] and 63°N [64], the cool and thermally restrictive climate during deposition of the Horseshoe Canyon and Wapiti formations occurred approximately 27–22 and 22–17 latitudinal degrees further south, respectively, than the temporally correlative PCF, reinforcing the apparent limitations on ectothermic animals in this polar ecosystem. In fact, the northernmost occurrence of any Late Cretaceous pseudosuchian fossil (according to the Paleobiology Database) is reported as a crocodylian osteoderm (TMP 1989.055.1604) from the Pipestone Creek Locality of the Wapiti Formation. (These data were downloaded from the Paleobiology Database on 14 August, 2025, using the group name ‘Pseudosuchia’.)

Finally, it is important to note that crocodyliforms are ‘numerical over-producers’ at the element-level, owing to a combination of variables, including a high tooth count, high and continuous tooth replacement rate and abundant osteoderms (all of which are diagnostic to the clade), in addition to their relatively large body size. As a result, crocodyliforms make up a significant component of Late Cretaceous vertebrate microsites from lower palaeolatitude sites in North America [13,15,65,66]. Given this taphonomic over-representation and high absolute abundance in southern assemblages, the complete lack of any diagnostic crocodyliform elements in the PCF most likely represents a true absence, not sampling bias.

### 4.5. Comparisons of the PCF teeth with avialans

Very few Late Cretaceous North American avialan specimens preserve teeth in jaws, with the record limited to only the hesperornithines *Hesperornis* and *Parahesperornis* and the ichthyornithine *Ichthyornis*, all from marine sediments [2,12,67]. The Late Cretaceous global record is not much better, expanding documented comparative material to only an unnamed enantiornithine from Brazil, represented by only two sets of premaxillae and an isolated dentary [42]. Plentiful isolated teeth are associated with the hesperornithine *Pasquiaornis*, but none are preserved within jaws [68]. Further, a single isolated tooth is described for the ichthyornithine *Janavis* from Belgium but is found in close enough association that it is almost certainly from *Janavis* [69]. This limited comparative record has only added to the confusion regarding the identity of putative avialan teeth.

The teeth of *Hesperornis* and *Ichthyornis* were described in detail by Dumont *et al.* [12]. *Hesperornis* teeth are both distally recurved and lingually curved, so much so that they appear ‘hooked’. They bear thin, relatively straight longitudinal ridges on their labial and lingual surfaces, thin mesial and distal carinae, and lack denticles. The teeth of *Parahesperornis* are similar, but smaller in size [2]. Teeth have also been described for *Pasquiaornis* from the Cenomanian of Saskatchewan but are not known from teeth in jaws. However, they are similar to the *Hesperornis* teeth. These teeth have mesiodistally broad carinae relative to *Hesperornis* and have enamel ornamentation on both the crown and carinae that closely matches that of the PCF avialan teeth [68,70]. In fact, the teeth of *Pasquiaornis* are more similar to PCF morphotypes 1–3 than any other observed avialan teeth, strongly supporting a hesperornithine identification (see especially RSM P2831.44, plate VII, figure 10, page 167 in Sanchez, [68]). It should be emphasized that the Bainbridge River locality from which these *Pasquiaornis* teeth derive is a marine deposit, with abundant avialan fossils, and no evidence of crocodyliforms or any other terrestrial taxa [68,71].

The teeth of *Ichthyornis* are essentially straight but become more recurved moving distally in the tooth row and are not lingually curved, making them easily distinguishable from the teeth of *Hesperornis*. The carinae are much wider mesiodistally than the thin carinae seen in *Hesperornis*, and they bear no enamel ornamentation. The tooth associated with *Janavis* is very similar to the distal teeth of *Ichthyornis* [12,69].

The record of Late Cretaceous enantiornithines known to possess teeth in jaws is extremely sparse [42,72]. Brazilian specimens of an unnamed taxon are among the best known and bear teeth that are distally recurved, not lingually curved, bear thin carinae and are unserrated [42]. They additionally do not bear any enamel ornamentation [42]. *Falcatakely* is known from a complete skull that bears only one small tooth in its premaxilla that was described as conical and unserrated [72]. There are no avialans known to have serrated teeth, and while the Early Cretaceous enantiornithine *Longipteryx* is known to have some larger teeth that bear crenulations on their distal margin, these are not homologous to the denticles of non-avialan theropods [73,74]. Additionally, while two enantiornithines from the Jehol Biota in China are known to bear teeth with longitudinal grooves on the lingual surfaces, none are known to have longitudinal ridges [75]. This may indicate that the presence of longitudinal ridges is a trait unique to ornithurine birds, especially considering the abundance of ornamented avialan teeth in ornithurine-dominant assemblages [7,8,22,31]. However, this should be treated with caution given the lack of Late Cretaceous enantiornithine material and the apparent absence of ridges in Ichthyornithes [12].

### 4.6. Potential sources of morphological variation in avialan teeth

The presence of seven morphotypes, distinguished based on crown curvature, recurvature, carina shape and enamel ornamentation, combine to create an avialan tooth sample with considerable overall disparity, enabling a number of testable hypotheses regarding the structure and palaeobiology of the PCF avialan assemblage. Morphological disparity produced by interspecific variation alone (implying homodont avialans with ontogeny not affecting tooth morphology) would indicate a higher richness of toothed birds within the PCF than is currently understood from skeletal material alone. It has been shown in *Ichthyornis* and *Hesperornis* that teeth become progressively more distally recurved moving towards the rostral end of the jaws, which may explain some of the variation in the PCF sample [12]. As *Ichthyornis* and *Hesperornis* both have edentulous premaxillae, it may be possible to infer that the most strongly recurved teeth are rostral dentary teeth. There is currently no evidence for variation in tooth lingual curvature in the jaws of *Hesperornis*, nor changes in ornamentation across the tooth row in *Hesperornis* or *Ichthyornis* [12,67]. Likewise, in this hypothesis, dental disparity could also indicate greater, unrecognized richness in more southerly formations than is understood based on skeletal remains alone [31]. Under this hypothesis, the relatively uniform sampling among the morphotypes may suggest similar relative abundance among taxa. This would have important implications for North American Late Cretaceous avialan diversity before the K–Pg extinction and subsequent Cenozoic radiation of birds, lending support to the idea that avialans continued to diversify before the extinction of non-avialan dinosaurs [8,22,76,77]. This richness, however, would not be enough to enable the survival of toothed birds, as studies have suggested that toothed maniraptorans would have been restricted to food chains dependent on living plant matter [19].

Conversely, if intraspecific sources of variation, including tooth row position, individual variation and ontogenetic change, are responsible for the PCF avialan tooth disparity, it would indicate that there are unrecognized, non-taxonomic sources of variation within avialan dentition. Based on the sizes of the strongly lingually curved PCF morphotypes 1, 2 and 3, there may be a size-dependent, and therefore potentially ontogenetic or tooth row position-based, signal of increased longitudinal ridge presence in larger teeth. Likewise, PCF morphotypes 5 and 7, bearing longitudinal ridges, are generally larger than the unornamented morphotype 6. This hypothesis may be supported by the Early Cretaceous enantiornithine *Longipteryx*, in which only large teeth bear crenulations on their distal margin [73,74]. Under this hypothesis, inferences of avialan diversity based on tooth morphotypes alone could overestimate true species richness. These hypotheses are directly testable by future discoveries of Late Cretaceous avialan teeth within jaws which can be assessed taxonomically, ontogenetically and serially.

### 4.7. Prince Creek Formation avialan richness

The PCF avialan assemblage consists of at least one species of ichthyornithine, at least one species of hesperornithine and at least two (but likely more) species of derived ornithurine (within or directly outside of Neornithes) [31]. Many of the isolated avialan teeth from the PCF are distinct from known teeth of both hesperornithines and ichthyornithines. The teeth of the ichthyornithines *Ichthyornis* and *Janavis* have a roughly straight crown and lack surface ornamentation [12,67,69]. *Hesperornis* teeth are typically strongly recurved, giving them a hooked appearance, have thin longitudinal ridges that run apicobasally, and defined, unornamented carinae that do not reach the apex or base of the tooth [12]. Morphotypes 1–3, as described here, are extremely similar to the described teeth of hesperornithines and may therefore belong to the PCF hesperornithine reported based on skeletal material [31,68]. As with hesperornithines, the teeth exhibit strong distal recurvature and lingual curvature giving them a hooked appearance. These teeth are nearly identical to the teeth described for *Pasquiaornis* [68], with some possessing thin longitudinal ridges on the surfaces of the crown and some also possessing ridges ornamenting broad carinae [68]. Variation in morphology in both the PCF and *Pasquiaornis* samples of teeth, including the presence of sulci, extent of carinae and enamel sculpturing, may be attributable to interspecific, intraspecific, tooth row or ontogenetic variation.

Morphotype 6, tall, labiolingually compressed teeth lacking longitudinal ridges, seems to be consistent with descriptions of teeth from both *Ichthyornis* and *Janavis*. PCF morphotypes 5 and 7 closely resemble this morphology but additionally bear thin, longitudinal ridges in varying combinations of presence across the labial and lingual surfaces as well as the carinae. Therefore, the PCF sample overlaps the known tooth condition for ichthyornithines but may include new combinations of crown shape and ornamental ridge presence.

In contrast to the other morphotypes described here which compare well to teeth known from within avialan jaws, morphotype 4 is currently not known from teeth within jaws and is therefore a strictly more ambiguous referral to Avialae. However, no other identification is currently better supported, given that the differential comparisons to the PCF non-avialan dinosaurs hold true for morphotype 4 as well. As nearly all of the PCF theropods are also known from perinatal teeth that resemble their adult tooth morphologies, we expect that a non-avialan theropod is not a likely identity for morphotype 4. Likewise, the absence of crocodyliforms from the PCF rules out a crocodyliform identity.

The PCF provides an opportunity to establish a baseline for avialan tooth diversity in a Late Cretaceous avialan assemblage without the confounding presence of crocodyliforms. Skeletal material supports the inference of an ornithurine-dominant fauna [31], with both edentulous and toothed forms present. Notably, skeletal remains of enantiornithines are completely absent from the PCF thus far, indicating that all PCF bird teeth are likely from ornithurines. Therefore, the presence of morphotypes like those described here within other Late Cretaceous terrestrial formations is consistent with the presence of ornithurines. It is therefore also possible that avialan teeth that do not match any of these morphotypes represent enantiornithine teeth. We caution, however, that as only one Late Cretaceous enantiornithine is known from teeth in jaws [42], teeth preserved within jaw bones are still desirable for definitive identification in other Late Cretaceous ecosystems.

### 4.8. Comparisons with Mohr *et al*.

Mohr *et al.* [23] examined 64 isolated teeth from the Belly River Group of Alberta, dividing them into 11 distinct morphotypes, only 1 of which was given reasonable support for an avialan attribution. If correct, this conclusion has extreme ramifications concerning our understanding of Mesozoic North American avialan diversity preceding the K–Pg mass extinction. Specifically, if most or all putative avialan teeth belong to crocodyliforms, Mohr *et al.* [23] suggest that the lack of bird teeth could be attributed to a largely edentulous ornithurine-dominated avialan assemblage. The avialan skeletal record from the Dinosaur Park Formation, from which many (*n* = 30, 47%) of the teeth, and nearly all morphotypes, assessed by Mohr *et al.* [23] originate, is indeed dominated by ornithurines [8,22,23].

Five of the seven tooth morphotypes we identified in the PCF overlap closely with morphotypes referred to as crocodyliforms by Mohr *et al.* [23] (table 4), though morphological variation occurs in each instance. Mohr *et al.*'s morphotypes 1 and 2 closely resemble PCF morphotype 7, though the Mohr *et al.* morphotype 1 bears broad and rounded longitudinal ridges (‘primary ridges’) which appear absent in any PCF teeth. Mohr *et al.*'s morphotype 3 closely resembles PCF morphotype 3, though none of the PCF morphotypes bear the irregular, wavy undulations splaying towards the carinae that ornament the teeth described by Mohr *et al.* [23]. Mohr *et al.*'s morphotype 7 (as figured in their fig. 2K) closely resembles PCF morphotype 4, though their written description of which teeth they collectively refer to their morphotype 7 do not all match the PCF morphotype 4. Mohr *et al.*'s morphotype 9 closely resembles PCF morphotype 6, although their lingually offset carinae do not match the PCF specimens. Considering the presence of these general tooth forms in the PCF, we propose that these tooth morphotypes in the Belly River Group may not represent crocodyliforms and instead may, at least in part, represent avialans (table 4).

**Table 4.** Comparison of the cf. Aves tooth morphotypes of Mohr *et al.* [23] with the PCF avialan tooth morphotypes of this study.

Mohr *et al.* [23] morphotype	PCF morphotype	Mohr *et al.* [23] ID	updated ID	notes
1	7	Crocodyliformes	Avialae	PCF lacks primary ridges
2	7	Crocodyliformes	Avialae	
3	3	Crocodyliformes	Avialae, Hesperornithes?	PCF lacks wavy ‘secondary ridges’
4	–	Crocodyliformes	–	
5	–	Crocodyliformes	–	
6	–	Crocodyliformes	–	
7	4	Crocodyliformes	Avialae	stated as being similar to Mohr *et al.* [23] morphotype 6
8	–	Crocodyliformes	–	
9	6	Crocodyliformes	Avialae, Ichthyornithes?	PCF morphotype may lack lingually offset carinae
10	–	Crocodyliformes	–	
11	–	Avialae	–	

The remaining tooth morphotypes (4–6, 8, 10–11) described by Mohr *et al.* [23] are currently absent from the PCF, leaving a crocodyliform identity possible. We refrain from broadly characterizing the teeth described by Mohr *et al.* [23] as definitively avialan, especially given the lack of teeth known from jaws, and because we do not claim to understand the sources of morphological variation within the PCF sample. Rather, our results open the possibility that some of the most similar morphotypes may be avialan, especially given the presence of derived ornithurine body fossils in the Belly River Group and the PCF [7,8,22,23,31].

## 5. Conclusion

Given the rarity of teeth rooted in jaws or associated with other identifiable skeletal data, it remains difficult to assess the true richness and diversity of Mesozoic avialans in the Late Cretaceous based on isolated teeth alone. The PCF provides a unique opportunity to better understand the morphological variation found in Late Cretaceous avialan dentition and validates this taxonomic assignment, given the absence of crocodyliforms from the ecosystem. In more confidently recognizing avialan teeth, we can better propose and test hypotheses relating to the biogeography, physiology and evolutionary history of Late Cretaceous birds.

## Supplementary Material



## Data Availability

All raw SEM images are accessible in the supplementary materials reposited at Zenodo [35]. Supplementary material is available online [78].
